# Hydrogen‐Associated Filling‐Controlled Mottronics Within Thermodynamically Metastable Vanadium Dioxide

**DOI:** 10.1002/advs.202414991

**Published:** 2025-02-14

**Authors:** Xuanchi Zhou, Yongjie Jiao, Wentian Lu, Jinjian Guo, Xiaohui Yao, Jiahui Ji, Guowei Zhou, Huihui Ji, Zhe Yuan, Xiaohong Xu

**Affiliations:** ^1^ Key Laboratory of Magnetic Molecules and Magnetic Information Materials of Ministry of Education & School of Chemistry and Materials Science Shanxi Normal University Taiyuan 030031 China; ^2^ Research Institute of Materials Science of Shanxi Normal University Collaborative Innovation Center for Advanced Permanent Magnetic Materials and Technology of Ministry of Education Taiyuan 030031 China; ^3^ Institute for Nanoelectronic Devices and Quantum Computing Fudan University Shanghai 200433 China; ^4^ Interdisciplinary Center for Theoretical Physics and Information Sciences Fudan University Shanghai 200433 China

**Keywords:** correlated oxides, hydrogenation, metal‐insulator transition, metastable material, topotactic phase modulation

## Abstract

The discovery of hydrogen‐associated topotactic phase modulations in correlated oxide system has emerged as a promising paradigm to explore exotic electronic states and physical functionality. Here hydrogen‐induced Mott phase transitions are demonstrated for metastable VO_2_ (B) toward new electron‐itinerant hydrogenated phases via introducing non‐equilibrium condition, delicately delivering a rich spectrum of hydrogen‐associated electronic states. Of particular interest, the highly robust but reversible hydrogenated phase achievable in metastable VO_2_ (B) significantly benefits protonic device applications, which is in contrast with well‐known VO_2_ (M1), where the metallic hydrogenated phase readily turns into insulating state with extensive hydrogen doping. Establishing correlated VO_2_ at metastable status fundamentally surpasses the thermodynamic restrictions to expand the adjustability in their electronic structure, giving rise to new electronic states and a superior resistive switching of 10^2^–10^5^ to the counterparts in widely‐reported VO_2_ (M1). Utilizing the theoretical calculations and synchrotron radiation analysis, the hydrogen‐associated phase modulation in metastable VO_2_ (B) is dominantly driven by band‐filling‐controlled orbital reconfiguration, while the concurrent structural evolution unveils a strong ion‐electron‐lattice coupling. The present work provides fundamentally new tuning knob for adjusting the energy landscape of electron‐correlated system, advancing the rational design of unachievable electronic states in hydrogen‐related equilibrium phase diagram.

## Graphical abstract

Hydrogen‐associated topotactic transition toward a new robust hydrogenated phase is uncovered in metastable VO_2_ via introducing non‐equilibrium conditions. Establishing correlated VO_2_ at metastable status extensively broadens the adjustability in electronic orbital configuration, enabling the rational design of exotic electronic states, but also elevates the driving force of the proton evolution for extensively tuning the energy landscape of electron‐correlated system.

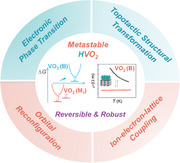

## 1 Introduction

Hydrogen‐associated filling‐controlled Mottronics provides a groundbreaking pathway to uncover new quantum states and exotic physical functionality within *d*‐orbital correlated oxides via adjusting the coupling between charge, lattice, orbital, and spin degrees of freedom.^[^ ^1^, ^2^, ^3^, ^4^, ^5^, ^6^, ^7^, ^8^, ^9^, ^10^, ^11^ ^]^ Beyond traditional tuning ways, proton evolution as enabled by using hydrogen spillover,^[^ ^12^, ^13^ ^]^ electrically tunable ionic liquid gating,^[^ ^3^ ^]^ or acid solution^[^ ^14^, ^15^, ^16^ ^]^ strategy opens up a new paradigm to enrich the structural, electronic, magnetic and optical phase diagram of correlated oxides. Over the past decade, hydrogen‐associated topotactic phase modulations have been reversibly achieved within an extensive collection of material system, covering VO_2_, *Re*NiO_3_, SrRuO_3_, SrCoO_2.5_, etc.^[^ ^17^, ^18^, ^19^, ^20^, ^21^, ^22^, ^23^, ^24^, ^25^, ^26^, ^27^, ^28^, ^29^ ^]^ Manipulating the ion‐electron‐lattice coupling in electron‐correlated system using hydrogenation enables the possibility in discovering new electronic states in the hydrogen‐related phase diagram.^[^ ^19^ ^]^ Typically, it is widely reported that hydrogenation triggers multiple orbital reconfigurations in correlated VO_2_ (M1) from correlated electronic ground state (*t*_2g_^1^ *e*_g_^0^) toward either electron‐itinerant state (*t*_2g_^1+Δ^ *e*_g_^0^)^[^ ^12^, ^30^, ^31^, ^32^, ^33^ ^]^ or electron‐localized state (*t*_2g_^2^ *e*_g_^0^),^[^ ^13^, ^14^, ^34^ ^]^ apart from conventional insulator‐metal transition (IMT) as triggered by changing of temperature.^[^ ^35^ ^]^ Utilizing the quantification of ^1^H concentration,^[^ ^36^ ^]^ the critical role of hydrogen in controlling the direction of opposite Mottronic transitions in VO_2_ (M1) is unveiled, in which a complete electron occupation in *d*_//_^*^ orbital arising from extensive hydrogen doping triggers the electron localization. This breakthrough significantly boosts interdisciplinary applications in neuromorphic learning,^[^ ^32^ ^]^ correlated electronics,^[^ ^30^ ^]^ sustainable energy conversions,^[^ ^34^, ^37^ ^]^ and magnetoelectronics.^[^ ^38^ ^]^

Such the multi‐step hydrogen‐associated Mott phase modulations unveil the rich physics and functionality within VO_2_ (M1), but inevitably face bottlenecks in protonic device applications due to a hydrogen‐concentration‐dependent phase transition route (Note S1, Supporting Information). One focal challenge lies in exploring more robust hydrogenated phases in correlated VO_2_ system through hydrogenation, which is yet fundamentally restricted by equilibrium phase diagram. Breaking the thermodynamic restrictions via introducing non‐equilibrium conditions provides tempting opportunities for harvesting exotic physical functionality and phenomenon, beyond conventional condensed matters. Establishing electron‐correlated system at their metastable status significantly adjusts the energy landscape of correlated system via surpassing the thermodynamic constraints, which also enlarges the driving force of proton evolution owing to an elevated Gibbs free energy (Δ*G*).^[^ ^39^, ^40^ ^]^ This endows the possibility in uncovering new electronic states that are unachievable in conventional hydrogen‐related equilibrium phase diagram. However, it is of great challenge to extend proton evolution to metastable material system for achieving topotactic phase modulations, without disturbing the lattice framework, owing to their destabilization nature. If analogous hydrogen‐associated Mott transitions of VO_2_ (M_1_) can be realized in metastable VO_2_ system, it will significantly extend hydrogen‐associated phase diagram, illuminating the path toward designing new quantum states in electron‐correlated system.

**Figure 1:**
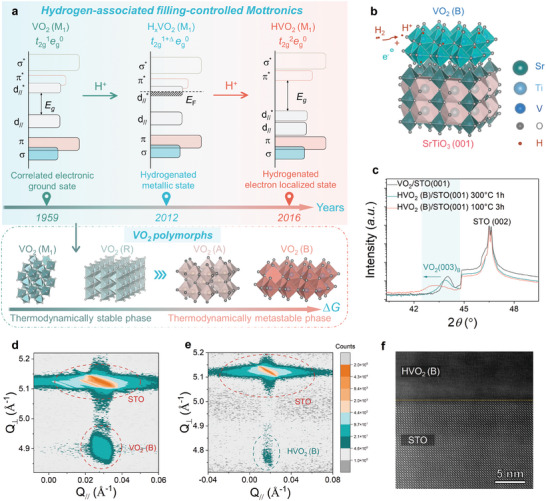
Structural transformation for VO_2_ (B) via proton evolution. a) Schematic illustration of the thermodynamically stable and metastable VO_2_ polymorphs, and the hydrogen‐associated multiple Mottronic transitions for VO_2_ (M_1_). b) Schematic diagram of as‐deposited VO_2_ (B)/STO (001) heterostructure. c) X‐ray diffraction (XRD) patterns as compared for VO_2_ (B)/STO (001) heterostructures upon various hydrogenation conditions. The respective reciprocal space mapping (RSM) as compared for VO_2_ (B)/STO (001) heterostructure d) before and e) after low‐temperature hydrogenation. f) The high‐angle annular dark‐field (HAADF) images of the interfacial regions for VO_2_/STO (001) heterostructure after hydrogenation at 100 °C for 3 h.

Here, we identified metastable VO_2_ as promising candidates to achieve proton evolution in metastable electron‐correlated system, delicately discovering new hydrogenated electronic states via introducing non‐equilibrium conditions. Apart from the well‐known VO_2_ (M1) phase, VO_2_ can exist in various polymorphic phases, among which metastable VO_2_ (B) holds great promise for achieving the hydrogenation, owing to its open framework (**Figure** **1**a; Figure S1, Supporting Information). The metastable VO_2_ (B) phase as formed by edge‐sharing and corner‐sharing VO_6_ octahedron exhibits an anisotropic layered structure associated with the stacking of V_4_O_10_‐type double layer.^[^ ^41^, ^42^, ^43^ ^]^ Due to an open lattice framework being capable of high tolerance and fast kinetics in ionic evolution, metastable VO_2_ (B) captures significant attention in energy storage.^[^ ^44^, ^45^, ^46^ ^]^ In addition, metastable VO_2_ (A) polymorph belonging to the P4_2_/nmc space group exhibits the high‐symmetry tetragonal crystalline structure and less distorted VO_6_ octahedron, which possesses a relatively enlarged forming energy, as compared with VO_2_ (B). In this work, we unveil hydrogen‐associated reversible orbital reconfiguration in metastable VO_2_ toward robust electron‐itinerant hydrogenated phases, which differs from the two‐step Mott phase modulations in VO_2_ (M_1_). This study is expected to open up a new paradigm to exploit exotic electronic state and functionality in electron‐correlated system that are unobtainable in conventional equilibrium phase diagram, advancing correlated electronic and iontronic device applications.

## 2 Results and Discussion

### 2.1 Hydrogen‐Associated Structural Evolution in Metastable VO_2_ (B)

Conventionally, hydrogenation can trigger multi‐step Mott phase modulations of correlated VO_2_ (M1) via directly manipulating the *d*‐orbital occupation and configuration. The introduced electrons tend to occupy the low‐energy *d*_//_^*^ orbital of VO_2_ (M1), triggering the metallization of VO_2_ (M1), while in contrast the integer filling in the *d*_//_^*^ orbital through extensive hydrogenation instead opens up a wider bandgap to result in the electron localization. Nevertheless, the challenge of realizing new robust electronic states in vanadium dioxide system through proton evolution lies in breaking the fundamental restrictions of thermodynamic equilibrium phase diagram. To address the above central issue, thermodynamically metastable VO_2_ (B) film with a layered structure was deposited on the single crystalline SrTiO_3_ (STO) (001) substrate upon an optimized condition using laser molecular beam epitaxy (LMBE) (Figure 1b; Figure S2, Supporting Information). It is worth noting that the lattice constant (*a*_0_) of the VO_2_ (B) film ($\frac{1}{3}$ *a*_0, film_ = 4.01 Å) is similar to the one for the STO substrate (*a*_0, sub._ = 3.905 Å), with a lattice mismatch of ≈‐2.6%. Therefore, the epitaxial template of STO substrate is expected to induce the interfacial heterogeneous nucleation that stabilizes the lattice framework of metastable VO_2_ (B) via overcoming the thermodynamic metastability. In addition, a relatively thin film thickness (e.g., ≈20 nm), together with a low depositing temperature (e.g., 400 °C), is herein selected to ensure the stabilization of the lattice framework for metastable VO_2_ (B). This understanding is confirmed by respective X‐ray diffraction (XRD) pattern of as‐deposited VO_2_ (B)/STO (001) heterostructure (Figure 1c), wherein the diffraction peak associated with the (003) plane of monoclinic VO_2_ (B) (e.g., 44.04°) appears adjacent to the STO substrate located at 46.5°.

To realize the proton evolution in metastable VO_2_ (B), the platinum (Pt) nano‐dots as the catalyst were sputtered onto the surface of as‐deposited VO_2_ (B)/STO hybrid, which can reduce the energy barrier for dissociating the hydrogen molecule into the protons and electrons at the triple‐phase boundary (Figure S3, Supporting Information).^[^ ^47^ ^]^ Considering the susceptible hydrogenation kinetics of VO_2_ to annealing conditions (Table S1, Supporting Information), the as‐grown Pt/VO_2_ (B)/STO heterostructures were hydrogenated at either 100 °C for 3 h or 300 °C for 1 h, according to our previous work.^[^ ^36^ ^]^ Upon low‐temperature hydrogenation, the diffraction peak associated with the (003) plane of VO_2_ (B) film shift leftward from 44.04° to 43.03°, while such the characteristic peak just shifts to 43.86° under a high‐temperature hydrogenation. As the variation in *c*‐axis lattice constant (*c*_0_) shown in Figure S4 (Supporting Information), a more pronounced lattice expansion as observed for VO_2_ (B) hydrogenated at a low temperature is consistent with previous understanding that a relatively mild hydrogenation condition is prone to introduce a higher hydrogen concentration.^[^ ^34^ ^]^ Such the hydrogenation process cannot destroy the expected crystallographic structure of metastable VO_2_ (B) but triggers a distinguished phase transformation toward a new hydrogenated phase, demonstrating hydrogen‐associated topotactic phase modulation.

Further consistency in hydrogen‐associated structural evolution of metastable VO_2_ (B) is demonstrated by respective reciprocal space mapping (RSM), as the results are shown in Figure 1d,e. The same *in‐plane* vector (e.g., *Q*_∥_) of VO_2_ (B) film and STO substrate demonstrates the coherent epitaxy of as‐fabricated VO_2_ (B)/STO heterostructure (Figure 1d). In addition, as‐observed enlarged *cross‐plane* vector (e.g., *Q*_⊥_) for the STO substrate with respect to the VO_2_ (B) film demonstrates an *in‐plane* biaxial compressive distortion in VO_2_ (B)/STO hybrid. After low‐temperature hydrogenation, the lattice of metastable VO_2_ (B) film is still in‐plane‐locked by the STO substrate as demonstrated by the same *in‐plane* vector *Q*_∥_ (Figure 1e). In addition, the lower magnitude of *Q*_⊥_ as observed for hydrogenated VO_2_ (B) (e.g., 4.774 Å^−1^) in comparison with the pristine one (e.g., 4.90 Å^−1^) indicates an *out‐of‐plane* lattice expansion of ≈2.5%, and the magnitude of which is similar to previous XRD result calculated by using Bragg's formula (≈2.2%). Furthermore, this result is also in accordance with the high‐angle annular dark‐field (HAADF) images of interfacial regions for hydrogenated VO_2_ (B)/STO hybrid (Figure 1f), wherein the extracted geometric phase analysis (GPA) profile also confirms an *out‐of‐plane* lattice strain (*ε*) with a lower *ε* along the *in‐plane* direction being observed (Figure S5, Supporting Information). Such the *out‐of‐plane* lattice expansion of metastable VO_2_ (B) via proton evolution is associated with the incorporated hydrogens that bond with the lattice oxygen to form the O─H interactions. In addition, the presence of hydrogen within the lattice of hydrogenated VO_2_ (B) is qualitatively characterized by using the time‐of‐flight secondary‐ion mass spectrometry (TOF‐SIMS) in Figure S6 (Supporting Information), in which a pronounced hydrogen concentration within hydrogenated VO_2_ (B) is clearly observed, in comparison with the STO substrate.

### 2.2 Electronic Phase Transition in Metastable VO_2_ (B) via Proton Evolution

**Figure 2:**
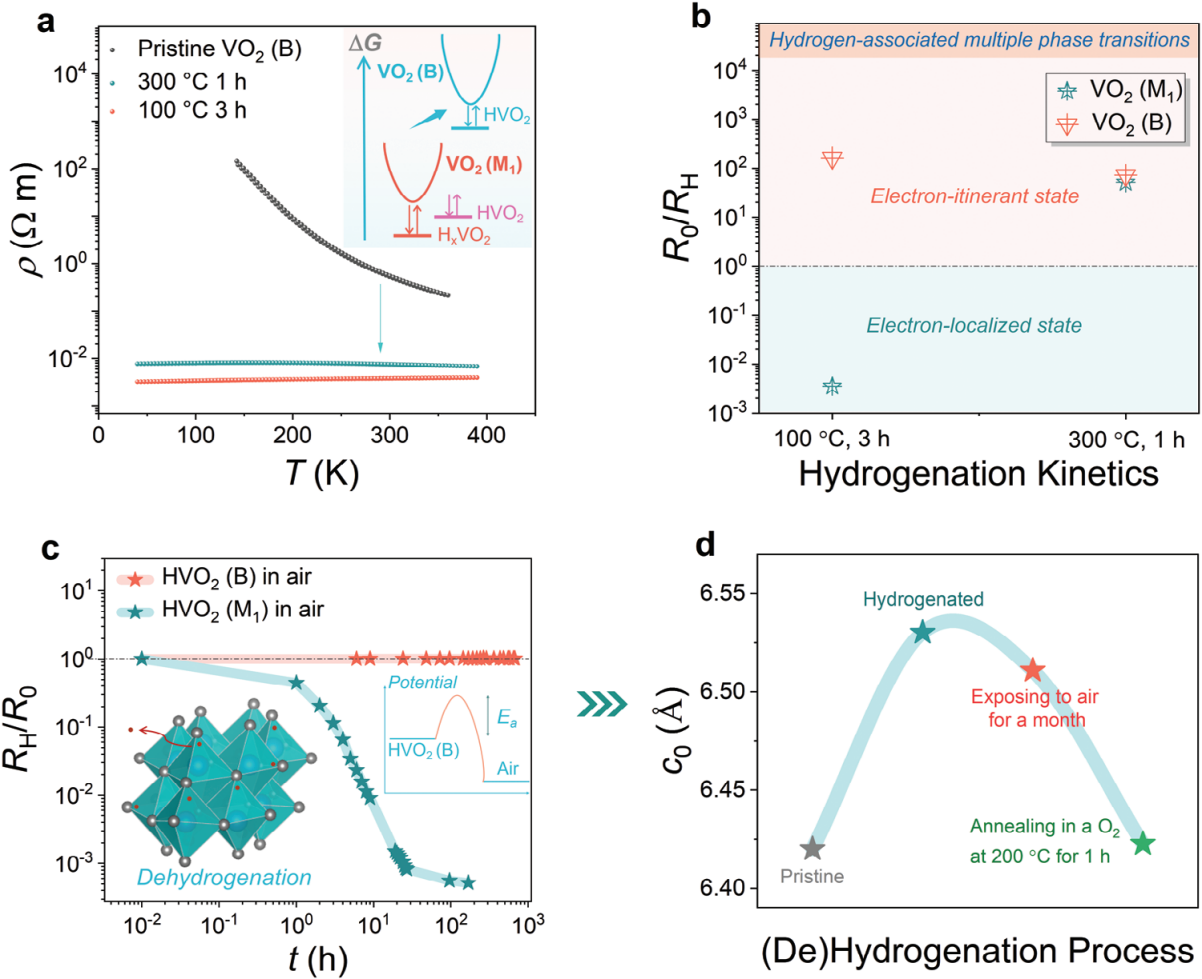
Hydrogen‐triggered electronic phase transitions for metastable VO_2_ (B). a) Temperature dependence of material resistivity (*ρ*‐*T*) as measured for the VO_2_ (B)/STO heterostructure upon various hydrogenation conditions. b) Hydrogen‐induced variation in the material resistivity (*R*_0_/*R*_H_) as compared for VO_2_ (B) and VO_2_ (M_1_)^[^ ^36^ ^]^ via hydrogenation. c) Variation in material resistivity when exposed VO_2_ (B) as hydrogenated at 100 °C for 3 h to the air, as compared with VO_2_ (M_1_).^[^ ^36^ ^]^ d) The variation in the *cross‐plane* lattice constant (e.g., *c*_0_) for VO_2_ (B) film upon (de)hydrogenation process.

In order to investigate hydrogen‐associated electronic phase transition, the temperature dependences of the resistivity (*ρ‐T*) are compared for the VO_2_ (B)/STO (001) heterostructures upon different hydrogenation conditions (**Figure** **2**a). Similar to previous report,^[^ ^44^ ^]^ pristine VO_2_ (B) exhibits a falling slope in its *ρ*‐*T* tendency, resembling typical transportation behavior of semiconductor. Performing the hydrogenation at whether 300 °C for 1 h or 100 °C for 3 h both results in a semiconductor‐metal transition in metastable VO_2_ (B), indicating a unified hydrogen‐related phase transition route, irrespective of hydrogenation conditions. This result starkly differs from the case of well‐known VO_2_ (M_1_), in which situation the electron‐itinerant hydrogenated phase based on *t*_2g_^1+Δ^ *e*_g_^0^ configuration transits to the electron‐localized phase based on *t*_2g_^2^ *e*_g_^0^ configuration with excessive hydrogen doping (Figure S7a, Supporting Information). It is well‐known that hydrogenation usually causes an *out‐of‐plane* lattice expansion of electron‐localized (electron‐itinerant) VO_2_ (M_1_) by ≈10% (≈2–3%).^[^ ^13^ ^]^ Therefore, the *out‐of‐plane* lattice expansion as herein achieved in metastable VO_2_ (B) (e.g., ≈2.2%) is also in accordance with the absence of hydrogen‐triggered electron localization. One possible reason is associated with the intrinsically thermodynamic metastability of VO_2_ (B) that impedes the extensive incorporation of hydrogens, forming a robust electron‐itinerant hydrogenated phase. Nevertheless, it is worth noting that the hydrogen‐triggered variation in the material resistance (e.g., *R*_0_/*R*_H_, where *R*_H_ denotes the resistance of hydrogenated phase) for VO_2_ (B) under low‐temperature hydrogenation exceeds the one hydrogenated at a higher temperature. Consistent with previous XRD result, performing the mild hydrogenation leads to a more robust regulation in the electrical transport property of metastable VO_2_ (B).

More importantly, in stark contrast to unstable electron‐localized HVO_2_ (M_1_),^[^ ^36^ ^]^ the electron‐itinerant hydrogenated phase as herein discovered in metastable VO_2_ (B) via proton evolution is rather robust when exposed to the air for a month, as demonstrated by the relatively stable *R*_0_/*R*_H_ evolution shown in Figure 2c. This understanding is further confirmed by corresponding XRD spectra (Note S2 and Figure S7b, Supporting Information), in which case the diffraction peak associated with hydrogenated phase cannot completely recover toward the pristine state under an ambient atmosphere for a month. The above results demonstrate a non‐volatile chemical stability of the newly‐discovered hydrogenated phase in metastable VO_2_ (B). However, annealing hydrogenated VO_2_ (B) at 200 °C for 1 h under an oxygen‐rich atmosphere (99% O_2_) results in a complete dehydrogenation process via overcoming the energy barrier for extracting intercalated hydrogens out, as indicated by the reversible recovery of hydrogenated phase in Figure 2d. Furthermore, the electrical transport property of VO_2_ (B) can reversibly recover toward the pristine state through dehydrogenation, in which the semiconducting transport property of pristine VO_2_ (B) can revive (Figure S7c, Supporting Information). Such the dehydrogenation process in metastable VO_2_ (B) can be reproduced well (Figure S8, Supporting Information). Further elevating the oxidizing annealing temperature to 350 °C instead triggers the decomposition of hydrogenated phase (Figures S6 and S7, Supporting Information), and this is related to the thermodynamically metastable nature of VO_2_ (B). The robust but reversible hydrogenated phase as newly discovered in metastable VO_2_ (B) via hydrogenation enriches the spectrum of electronic states in correlated VO_2_ system, which benefits protonic device applications.

### 2.3 Chemical Environment and Hydrogen Absorption of VO_2_ (B) Upon Hydrogenation

**Figure 3:**
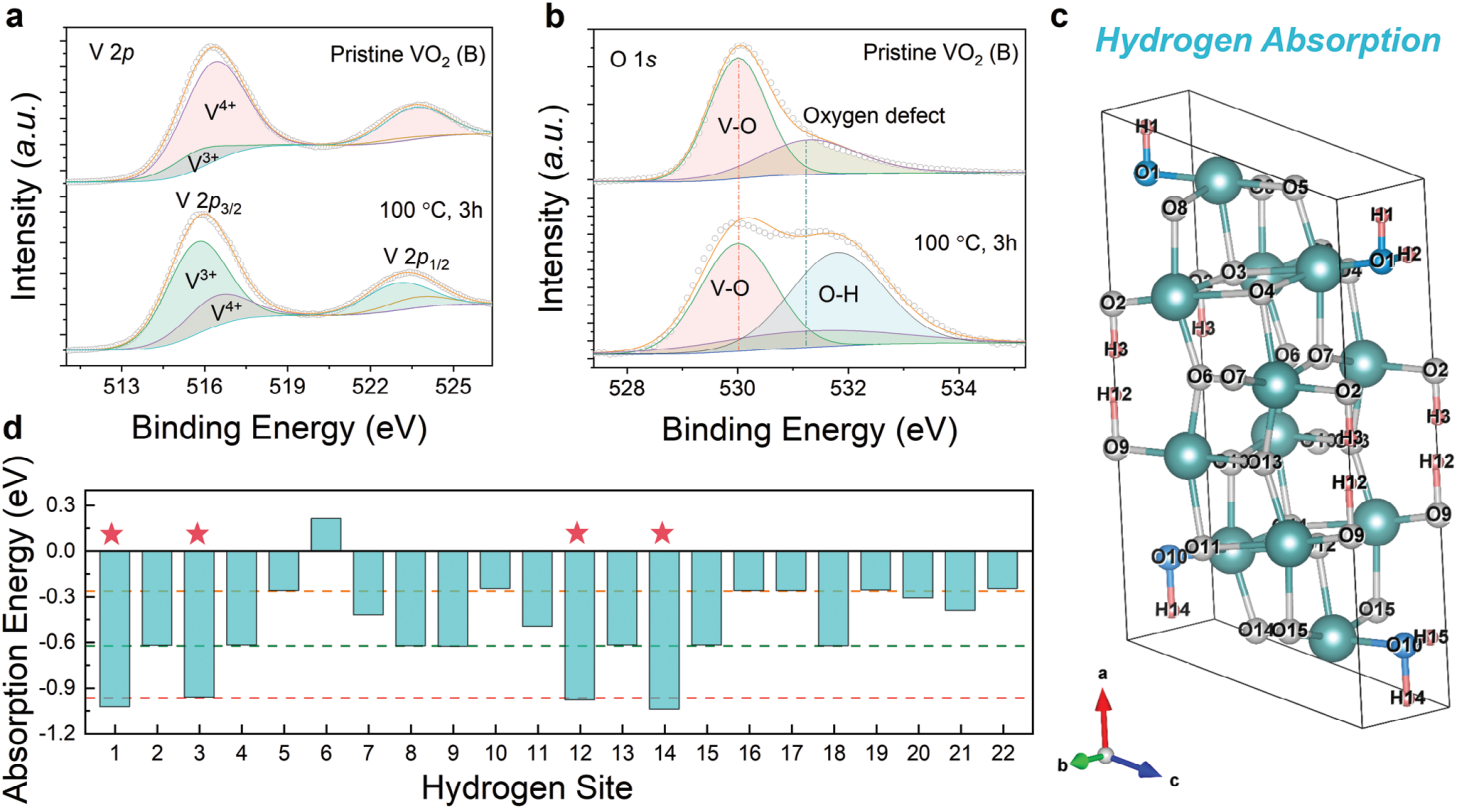
Chemical environment and hydrogen absorption within hydrogenated VO_2_ (B). a,b, The X‐ray photoelectron spectra (XPS) for the core levels of a) vanadium and b) oxygen of VO_2_ (B)/STO (001) heterostructure as hydrogenated 100 °C for 3 h. c) Schematic of hydrogen absorption sites for hydrogenated VO_2_ (B). d) Calculated absorption energies of hydrogenated VO_2_ (B) by using first‐principles calculations.

To probe the variations in the valence state and chemical environment for metastable VO_2_ (B) upon hydrogenation, X‐ray photoelectron spectra (XPS) analysis was performed, as the V 2*p* and O 1*s* core‐level peaks shown in **Figure** **3**a,b, respectively. Noting similar electron‐itinerant state under different hydrogenation conditions, the low‐temperature hydrogenated VO_2_ (B) with a more pronounced *R*_0_/*R*_H_ regulation was deeply investigated by using the XPS analysis. Hydrogenation results in a reduction in the valence state of vanadium from V^4+^ toward V^3+^, as demonstrated by the V 2*p* core‐level peak (Figure 3a). In the spectrum of O 1*s* core‐level, the characteristic peak representing the lattice oxygen is observed to be located at ≈530 eV, while the adjacent peak located at ≈531 eV corresponds to the oxygen defects as in situ formed in the deposition of metastable VO_2_ (B) film. Performing the hydrogenation results in an elevation in the relative intensity of O─H bond (e.g., ≈531.8 eV) for metastable VO_2_ (B) with respect to V─O bond, indicating that the incorporated hydrogens tend to bond with the lattice oxygen to form the O─H interactions (Figure 3b).

To investigate the preferential occupied site for hydrogen in the lattice of metastable VO_2_ (B), first‐principles calculations based on density functional theory (DFT) were performed by occupying one hydrogen atom to the non‐equivalent oxygen atom site in a 1 × 1 × 1 VO_2_ (B) unit cell. The Hubbard on‐site repulsion *U* of 3.8 eV has been used to account for the strong correlation of VO_2_. As the configuration of possible twenty‐two distinct hydrogen absorption positions illustrated in Figure S9 (Supporting Information), the respective hydrogen absorption energies are obtained by fully relaxing the atomic coordinates (Figure 3c,d). It is found that hydrogen adsorption is more energetically favorable at the H‐1, H‐3, H‐12, and H‐14 sites that are aligned along the *a*‐axis direction of the monoclinic VO_2_ (B), bonding with the oxygen at a relative empty site. Analogous to the preferential diffusion pathway along the empty *c*‐axis of rutile VO_2_ (M1),^[^ ^48^ ^]^ equivalent to the *a*‐axis of the monoclinic VO_2_ (M1), intercalated protons energetically bond with the oxygen in the monoclinic VO_2_ (B) along the empty *a*_M_‐axis channel. Such the similar hydrogenation kinetics is associated with an enlarged energy barrier for hydrogen diffusion along the *c*‐axis direction of monoclinic VO_2_ owing to the repulsion between the proton and vanadium ion, which differs from a helical diffusion of hydrogens in the oxygen ions along the [100]_M_ or [001]_R_ direction.

### 2.4 Band Structure for Metastable VO_2_ (B) via Proton Evolution

**Figure 4:**
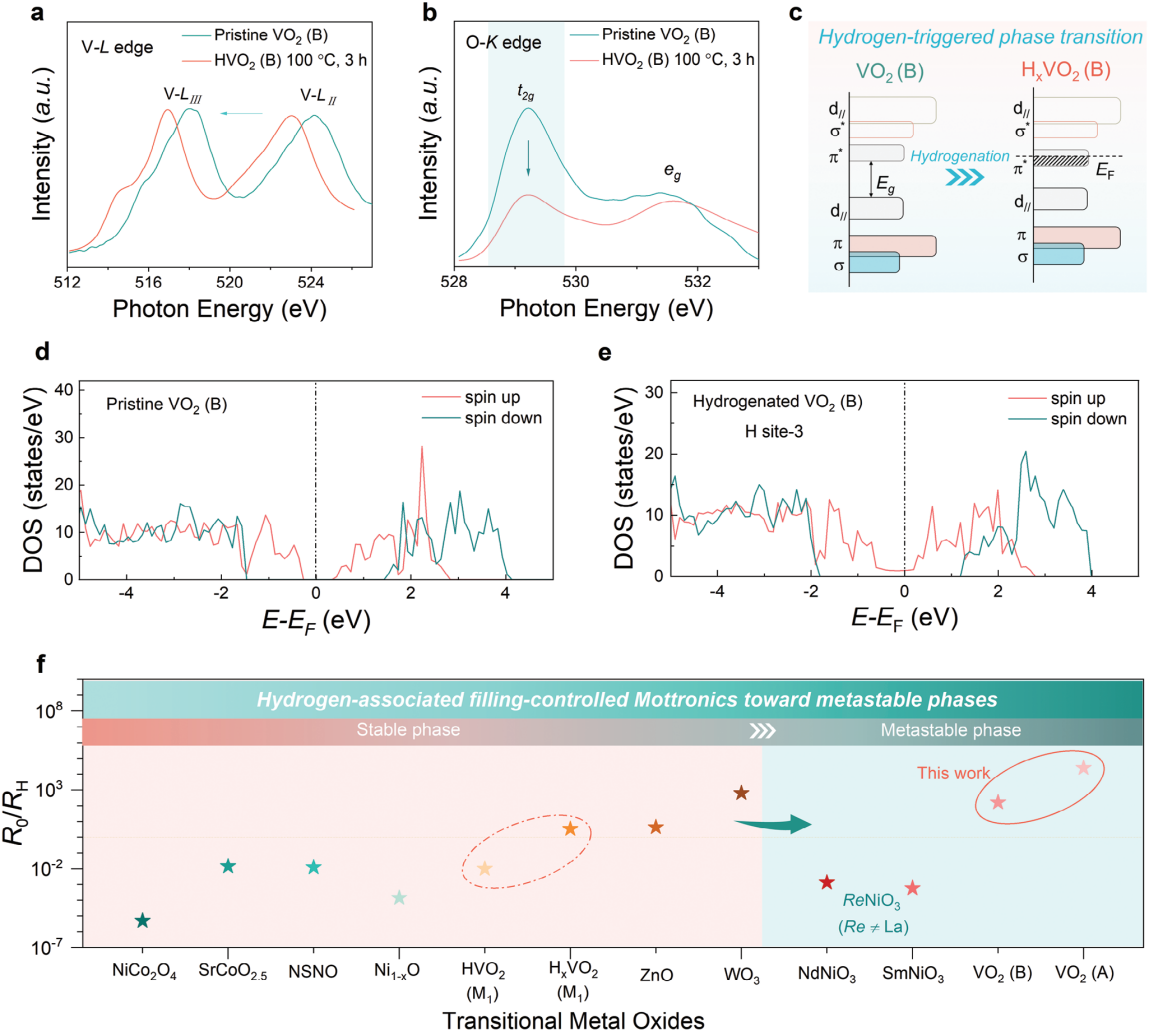
Electronic structure for metastable VO_2_ upon hydrogenation. a,b, The near edge X‐ray absorption fine structure (NEXAFS) spectra for the a) V‐*L* edge and b) O‐*K* edge of VO_2_ (B)/STO (001) heterostructure before and after low‐temperature hydrogenation. c) Schematic of hydrogen‐induced variation in the electronic orbital configuration of VO_2_ (B). d,e, Calculated density of states (DOS) of VO_2_ (B)/STO heterostructure d) before and e) after hydrogenation. f) The presently achieved *R*_0_/*R*_H_ for metastable VO_2_ polymorphs, as compared with other typical transitional metal oxides.^[^ ^13^, ^19^, ^51^, ^52^, ^53^ ^]^

Further consistency observed from the vanadium valence state for hydrogenated VO_2_ (B) is demonstrated by the V‐*L* edge spectrum, as characterized by the near‐edge X‐ray absorption fine structure (NEXAFS) analysis (**Figure** **4**a). Conventionally, the V‐*L*_III_ peak located at ≈518 eV in the V‐*L* edge spectrum is associated with the V 2*p*_3/2_ → 3*d* transition, while the V‐*L*_II_ peak (≈524 eV) reflects the V 2*p*_1/2_ → 3*d* transition. Consistent with the aforementioned XPS result, both the V‐*L*_III_ and the V‐*L*_II_ peaks for metastable VO_2_ (B) shift leftward upon hydrogenation, indicating a reduction in the valence state of vanadium from V^4+^ to V^(4‐δ)+^. Considering the hybridization between the V‐3*d* and O‐2*p* orbitals and empty O‐2*p* states, the O 1*s* core‐level peak associated with O 1*s* → 2*p* transition qualitatively represents the electron occupation in the V‐3*d* orbital, providing more information associated with the band structure of metastable VO_2_ (B).^[^ ^49^ ^]^ Therefore, the reduction in the relative intensity of the first peak in the O 1*s* spectrum indicates the electron filling in the low‐energy *t*_2g_ band of VO_2_ as formed by V‐3*d*_xz_, 3*d*_yz_, and 3$d_{x^{2}-y^{2}}$ orbitals, as compared with the second peak that corresponds to the *e*_g_ band formed by V‐3*d*_xy_ and 3$d_{z^{2}-r^{2}}$ orbitals (Figure 4b; Figure S10, Supporting Information). On the basis of electronic orbital configuration for metastable VO_2_ (B), the electrons as released by hydrogenation are recognized to occupy the low‐energy *π* ^*^ orbital of VO_2_ (B) belonging to the *t*_2g_ band and elevate the Fermi level (*E*_F_), uncovering the electron‐doping‐mediated Mott transition (Figure 4c).

In order to gain a deep insight for the underlying mechanism that drives the hydrogen‐associated topotactic transition within metastable VO_2_ (B), the spin‐polarized density of states (DOS) was calculated (Note S3, Supporting Information; Figure 4d,e). Consistent with the experimentally observed semiconducting ground state, a band gap of ≈0.6 eV is observed for the case of pristine VO_2_ (B) (Figure 4d). Nevertheless, adding up one hydrogen at an energetically favorable absorption position in the lattice of VO_2_ (B) (e.g., H‐3 site) results in a non‐vanishing DOS near the *E*_F_ that is dominated by the spin‐up electrons in the V‐3*d* orbital (Figure 4e). Analogously, the hydrogen‐triggered metallization of VO_2_ (B) is demonstrated by calculating the corresponding band structures (Figures S11 and S12, Supporting Information). In addition, such the finite DOS near the *E*_F_ can be also observed when adding up one hydrogen atom at the other hydrogen absorption sites with different absorption energies (Figures S13–S15, Supporting Information). Utilizing the DFT calculations, the electron carriers as introduced by proton evolution tend to occupy the low‐energy empty states in the conduction band of VO_2_ (B) that are shifted downward to the *E*_F_, participating in the carrier conduction. Therefore, the underlying mechanism driving the hydrogen‐associated Mott phase modulation for VO_2_ (B) is identified to be the *d*‐band filling using electron carriers.

Introducing non‐equilibrium conditions to the electron‐correlated system enables the possibility in designing unobtainable electronic states under equilibrium conditions, endowing tempting opportunities to explore exotic physical functionality and phenomenon. Here establishing correlated VO_2_ at metastable status fundamentally surpasses the thermodynamic restrictions to extensively tune the energy landscape of correlated system, discovering new hydrogenated phases (Figure 4f). Without loss of generality, proton evolution is extended to metastable tetragonal VO_2_ (A) with a less distorted VO_6_ octahedron and relatively low Δ*G*, analogously triggering topotactic phase modulation toward new hydrogenated phase (Note S4, Supporting Information; Figures S16–S19, Supporting Information). The hydrogen‐induced resistive switching (*R*_0_/*R*_H_) of exceeding 10^5^ presently achievable in metastable VO_2_ (A) outperforms the counterparts in well‐known VO_2_ (M1) by almost two orders of magnitude, benefiting potential protonic device application in correlated electronics (Figure S20 and Table S2, Supporting Information). In addition, the high tolerance in the ionic evolution of metastable VO_2_ (B) related to its open framework, as well as the hydrogen‐associated reversible phase modulation, hints at great potential in the field of energy conversions. To the best of our knowledge, apart from the widely‐reported VO_2_ (M1), it is the first time that other robust hydrogenated phases can be realized in vanadium dioxide through proton evolution, resulting in a rich spectrum of electronic states in this electron‐correlated system. Remarkably, hydrogen‐associated phase modulation in metastable VO_2_ toward robust electron‐itinerant state strongly differs from the two‐step insulator (VO_2_)‐metal (H_x_VO_2_)‐highly insulator (HVO_2_) transition in well‐known VO_2_ (M1) through hydrogenation. Such the hydrogen‐induced electronic states achievable in metastable VO_2_ are not only rather robust and reversible, irrespective of incorporated hydrogen concentration, but also give rise to a giant resistive switching superior to the VO_2_ (M1), which benefits protonic device applications. The key point that realizes hydrogen‐associated topotactic phase modulation in metastable material system, without perturbing their lattice framework, hinges on a suitable Δ*G* and open framework, overcoming the trade‐off between non‐equilibrium state and topotactic transition. On this basis, assisted by high‐throughput calculations, more potential candidates for realizing proton evolution are envisioned to be found in metastable electron‐correlated system. Beyond that, the hydrogen aggregation around a defect‐rich region as induced by hydrogen interactions can analogously bring in non‐equilibrium conditions to electron‐correlated system. Consequently, an artificial design of material microstructure via engineering the boundary configuration also sheds lights on exploring new hydrogenated phases arising from non‐equilibrium states in electron‐correlated system. In the future, more promising protonic device applications using the hydrogen‐associated phase modulation of metastable VO_2_ are envisioned to be further explored, especially for the applications in the field of correlated electronics, artificial intelligence, and energy conversions.

## 3 Conclusion

In summary, we presented a promising design strategy to access new electronic states and physical functionalities in hydrogenated electron‐correlated system, beyond conventional equilibrium phase diagram. Hydrogen‐induced topotactic phase modulations toward new electron‐itinerant states were realized in correlated VO_2_ system via overcoming thermodynamic restrictions by introducing non‐equilibrium states. A reversible hydrogen‐associated phase transition route in metastable VO_2_ (B) toward robust hydrogenated phase is in contrast with the multi‐step phase modulations in well‐known VO_2_ (M1) depending on incorporated hydrogen concentration, which overcomes the bottlenecks in protonic device applications. In addition, the hydrogen‐induced resistive switching of 10^2^–10^5^ achievable in metastable VO_2_ system outperforms the counterparts in VO_2_ (M1). Utilizing the NEXAFS analysis and DFT calculations, the underlying mechanism driving the phase transition for metastable VO_2_ (B) is identified to be hydrogen‐associated electron‐doping process that reconfigures its *d*‐orbital band structure. Combined with a topotactic structure evolution of VO_2_ (B) arising from O─H interactions, an intriguing ion‐electron‐lattice coupling is unveiled in this metastable electron‐correlated system, which serves as an emerging platform for probing rich correlated physics. This study provides a new guideline for rationally designing unachievable electronic states in traditional equilibrium phase diagram of electron‐correlated system, which offers vast possibilities to explore exotic physical functionality and phenomenon.

## 4 Experimental Section

### Fabrication of As‐Deposited VO_2_ Heterostructures

The metastable VO_2_ (B) films were deposited on the single crystalline SrTiO_3_ (001) substrates via the laser molecular beam epitaxy (LMBE). The deposition temperature, the oxygen pressure, the target‐substrate distance, and the laser fluence were set to 400 °C, 2.0 Pa, 45 mm, and 1.0 J cm^2^, respectively. Afterward, the as‐deposited VO_2_ (B) films were naturally cooled down to the room temperature upon the same oxygen partial pressure. Prior to the hydrogenation, the platinum dots with a ≈20 nm thickness were sputtered onto the surface of as‐deposited VO_2_ (B) heterostructure. Finally, on the basis of the hydrogen spillover strategy, as‐made VO_2_ (B) films were annealed in a H_2_/Ar gas mixture for achieving effective hydrogenation process, according to the previous work.^[^ ^36^ ^]^

### Material Characterizations

The crystal structures of VO_2_ (B) films were characterized by using the X‐ray diffraction (XRD) and reciprocal space mapping (RSM) (Rigaku, Ultima IV). The X‐ray photoelectron spectroscopy (XPS) (Thermo, K‐Alpha X) was used to probe the variations in the chemical environment of hydrogenated VO_2_ (B) films, while respective electronic structures were investigated by using the near edge X‐ray absorption fine structure (NEXAFS), as performed in the beam line BL08U1A at the Shanghai Synchrotron Radiation Facility (SSRF). Temperature dependent resistivity of the VO_2_ (B) films were measured by using a commercial physical property measurement system (PPMS) (Quantum design), and the room‐temperature resistance measurements were performed by using a Keithley 2400 system.

### First‐Principles Calculations

The first‐principles calculations were performed by using a plane‐wave ultrasoft pseudopotential method as implemented in the QUANTUM ESPRESSO. An energy cutoff of 70 Ry for the plane‐wave expansion, Monkhorst‐Pack grid of 9 × 27 × 16 for k‐point sampling, and generalized gradient approximation (GGA) + *U* were herein used.^[^ ^50^ ^]^ In addition, spin‐polarized DFT calculations were employed, while the Hubbard *U* value for correlated V‐3*d* states of VO_2_ (B) is set to be 3.8 eV. The band structure of hydrogenated VO_2_ (B) is investigated by inserting one H atom per unit cell (H_0.125_VO_2_). The atomic coordinators were fully relaxed with the force tolerance of 10^−3^ Ry/Bohr. In the self‐consistent calculation, the energy convergence criterion was set to be 10^−8^ Ry. To determine the hydrogen absorption energy at a possible interstitial binding position within the lattice of VO_2_ (B), the total energy differences between pristine and hydrogenated VO_2_ (B) were calculated $E_{abs.}=E_{\mathrm{HV}O_{2}}-E_{VO_{2}}-\frac{1}{2}E_{H_{2}}$.

## Conflict of Interest

The authors declare no conflict of interest.

## Author Contributions

X.Z. conceived this study, and lead the project; X.Z., X.X., and G.Z. supervised the study; X.Z. planned for the experiment, analyzed the results, and carried out the partial sample measurements; Y.J., X.Y, and J.J. grew VO_2_ films, and performed hydrogenation experiments under the supervision of X.Z. and X.X.; W.L. performed the DFT calculations assisted by Z.Y.; J.G. performed the TEM analysis; X.X., G.Z., and H.J. provided the supports in the sample prepares and transport measurements; X.Z. wrote the paper with contributions from all authors; All authors discussed the results and commented on the final manuscript.

## Supporting information



Supporting Information

## Data Availability

The data that support the findings of this study are available from the corresponding author upon reasonable request.
