# Supplementary material for: Hydrogen‐Associated Filling‐Controlled Mottronics Within Thermodynamically Metastable Vanadium Dioxide
Source: Adv Sci (Weinh). 2025 Feb 14;12(14):2414991. doi: 10.1002/advs.202414991 (PMC11984838; doi:10.1002/advs.202414991)
Supplement: Supplementary file 1 — Supporting Information [file ADVS-12-2414991-s001.docx]

Supporting Information

**Hydrogen-associated filling-controlled Mottronics within thermodynamically metastable vanadium dioxide**

*Xuanchi Zhou^*^, Yongjie Jiao, Wentian Lu, Jinjian Guo, Xiaohui Yao, Jiahui Ji, Guowei Zhou^*^, Huihui Ji, Zhe Yuan, Xiaohong Xu^*^*

Prof. X.C. Zhou, Y.J. Jiao, Dr. W.T. Lu, Dr. J.J. Guo, X.H. Yao, J.H. Ji, Prof. G.W. Zhou, Dr. H.H. Ji, Prof. X.H. Xu

Key Laboratory of Magnetic Molecules and Magnetic Information Materials of Ministry of Education & School of Chemistry and Materials Science, Shanxi Normal University, Taiyuan, 030031, China

E-mail: [xuanchizhou@sxnu.edu.cn](mailto:xuanchizhou@sxnu.edu.cn), [zhougw@sxnu.edu.cn](mailto:zhougw@sxnu.edu.cn), [xuxh@sxnu.edu.cn](mailto:xuxh@sxnu.edu.cn)

Prof. X.C. Zhou, Dr. W.T. Lu, Prof. G.W. Zhou, Dr. H.H. Ji, Prof. X.H. Xu

Research Institute of Materials Science of Shanxi Normal University, Collaborative Innovation Center for Advanced Permanent Magnetic Materials and Technology of Ministry of Education, Taiyuan 030031, China

Prof. Z. Yuan

Institute for Nanoelectronic Devices and Quantum Computing, Fudan University, Shanghai, 200433, China

Prof. Z. Yuan

Interdisciplinary Center for Theoretical Physics and Information Sciences, Fudan University, Shanghai 200433, China

**This file includes:**

Supplementary Notes 1-4, Supplementary Figures 1-20, Supplementary Tables 1-2 and Supplementary References.

**Contents**

**Supplementary Note 1. Filling-controlled Mottronics within VO_2_.**

**Figure S1. Schematic of electronic phase transitions within VO_2_**

**Figure S2. AFM and XRD spectra for metastable VO_2_ (B)**

**Figure S3. Schematic illustration of as-used hydrogen spillover method**

**Supplementary Table 1. Hydrogenation kinetics for VO_2_ using hydrogen spillover strategy**

**Figure S4. Lattice expansion for VO_2_ (B) upon hydrogenation**

**Figure S5. GPA analysis for hydrogenated VO_2_ (B)**

**Figure S6. TOF-SIMS measurement for hydrogenated VO_2_ (B)**

**Figure S7. Reversible phase transition of VO_2_ (B) via hydrogen evolution**

**Supplementary Note 2. Chemical stability for hydrogenated VO_2_ (B) phase**

**Figure S8. Chemical stability for hydrogenated VO_2_ (B)**

**Figure S9. Schematic of the hydrogen absorption in VO_2_ (B)**

**Figure S10. Electron filling in the band structure of VO_2_ (B)**

**Supplementary Note 3. DFT calculations for hydrogenated VO_2_ (B)**

**Figures S11-S12. Band structure for VO_2_ (B) upon hydrogenation**

**Figures S13-S15. Calculated density of states of hydrogenated VO_2_ (B)**

**Supplementary Note 4. Hydrogen-associated phase modulation within metastable VO_2_ (A)**

**Figure S16. Crystalline structure and transport property for VO_2_ (A)**

**Figures S17-S18. Hydrogen-associated phase modulation for VO_2_ (A)**

**Figure S19. XPS spectra for VO_2_ (B) via hydrogen evolution**

**Figure S20. Hydrogen-triggered resistive regulation within VO_2_.**

**Supplementary Table 2. Hydrogen-associated phase transitions within transitional metal oxides**

**Supplementary References**

**Supplementary Note 1. Filling-controlled Mottronics within VO_2_.**

Previously, hydrogenation is recognized to directly donate electron carriers into the conduction band of traditional *n*(*p*)-type semiconductors, where the material conductivity is linearly elevated (suppressed) with carrier concentration, as demonstrated in WO_3_, TiO_2_ and ZnO.^[1-6]^ Apart from conventional semiconductors, hydrogen-associated electron-doping process can reconfigure the band structure of correlated oxides to trigger inter-atomic Coulomb repulsions, abruptly adjusting the physical property. Notably examples include correlated VO_2_, *Re*NiO_3_, and La_0.67_Sr_0.33_MnO_3_, which extensively enriches their hydrogen-associated structural, electronic and magnetic phase diagram via manipulating the ion-electron-phonon-spin interactions.^[7-10]^ Typically, hydrogen-associated filling-controlled Mottronics opens up a new paradigm to trigger multiple topotactic phase transitions within stable VO_2_ (M_1_) ^[11-12]^ that advances abundant device applications,^[13-14]^ beyond critical-temperature-triggered insulator-metal transition (Figure S1).^[15-17]^ The term Mottronics denotes an emerging electron technology exploiting the Mott transition from itinerant to localized electronic behaviour in correlated electron systems. Performing the low-temperature (high-temperature) hydrogenation for a long (short) period under a H_2_/Ar gas mixture triggers the formation of electron-localized (electron-itinerant) hydrogenated phase within VO_2_ (M_1_), as more details summarized in Table S1. Such the multi-step phase modulations in VO_2_ (M_1_) are associated with the incorporated hydrogen concentration that determinates the direction of hydrogen-related orbital reconfiguration. Nevertheless, to date, the proton evolution is still restricted in thermodynamic equilibrium phase diagram, fundamentally impeding the discovery of exotic physical functionality. From one aspect, establishing the correlated oxides at metastable status can overcome the basic limitation associated with the thermodynamic equilibrium conditions. Moreover, the thermodynamic metastability arising from an enlarged Δ*G* is demonstrated to enhance the driving force associated to the hydrogenation process that enables the possibility in regulating the physical property at a more robust energy scale.^[18]^ From the other aspect, metastable correlated oxides is prone to be decomposed or chemically reduced via hydrogen evolution, which deviates from the expected hydrogen-related topotactic transition. Notable example including metastable *Re*NiO_3_ with heavy rare-earth composition exemplifies the challenge of achieving proton evolution in metastable material system, which is readily decomposed into NiO and *Re*_2_O_3_ upon hydrogenation owing to a highly distorted NiO_6_ octahedron. However, an open framework associated with the layered structure endows metastable VO_2_ (B) with the potential of realizing the proton evolution. In contrast to the commonly utilized solid-state ^[19]^ or solution-phase reaction,^[20-21]^ interfacial heterogeneous nucleation using SrTiO_3_ epitaxial substrate ^[22-26]^ provides a convenient pathway to overcome its intrinsic metastability for depositing VO_2_ (B) film and probing associated proton evolution. Therefore, whether such the hydrogenation can be extended to metastable VO_2_ (B) polymorph, accessing more exotic electron phases and functionality, is still a critical open question worthy of further exploration.


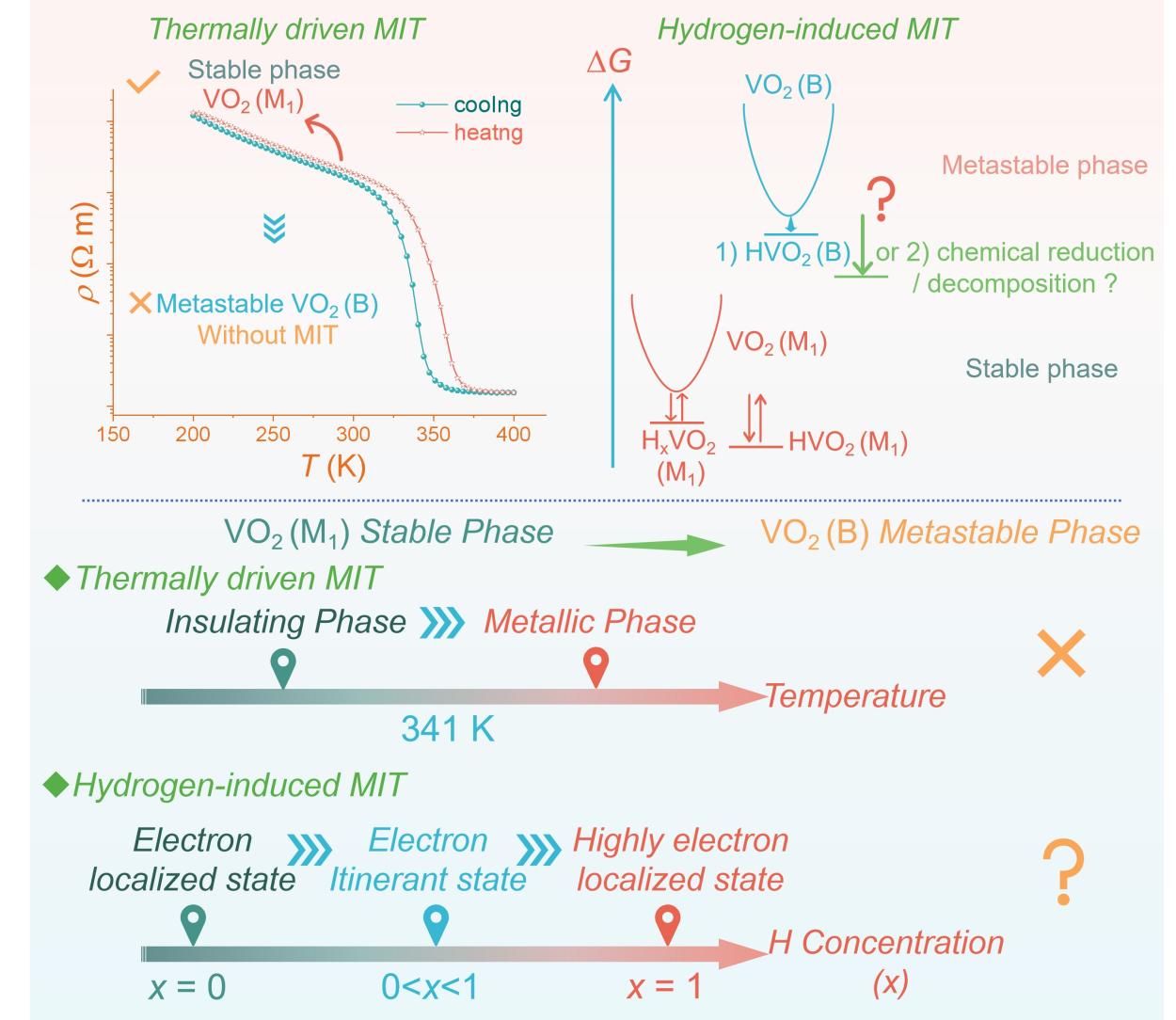


**Supplementary Figure 1.** Schematic of multiple electronic phase transitions within vanadium dioxide system as triggered by the critical temperature or hydrogenation.


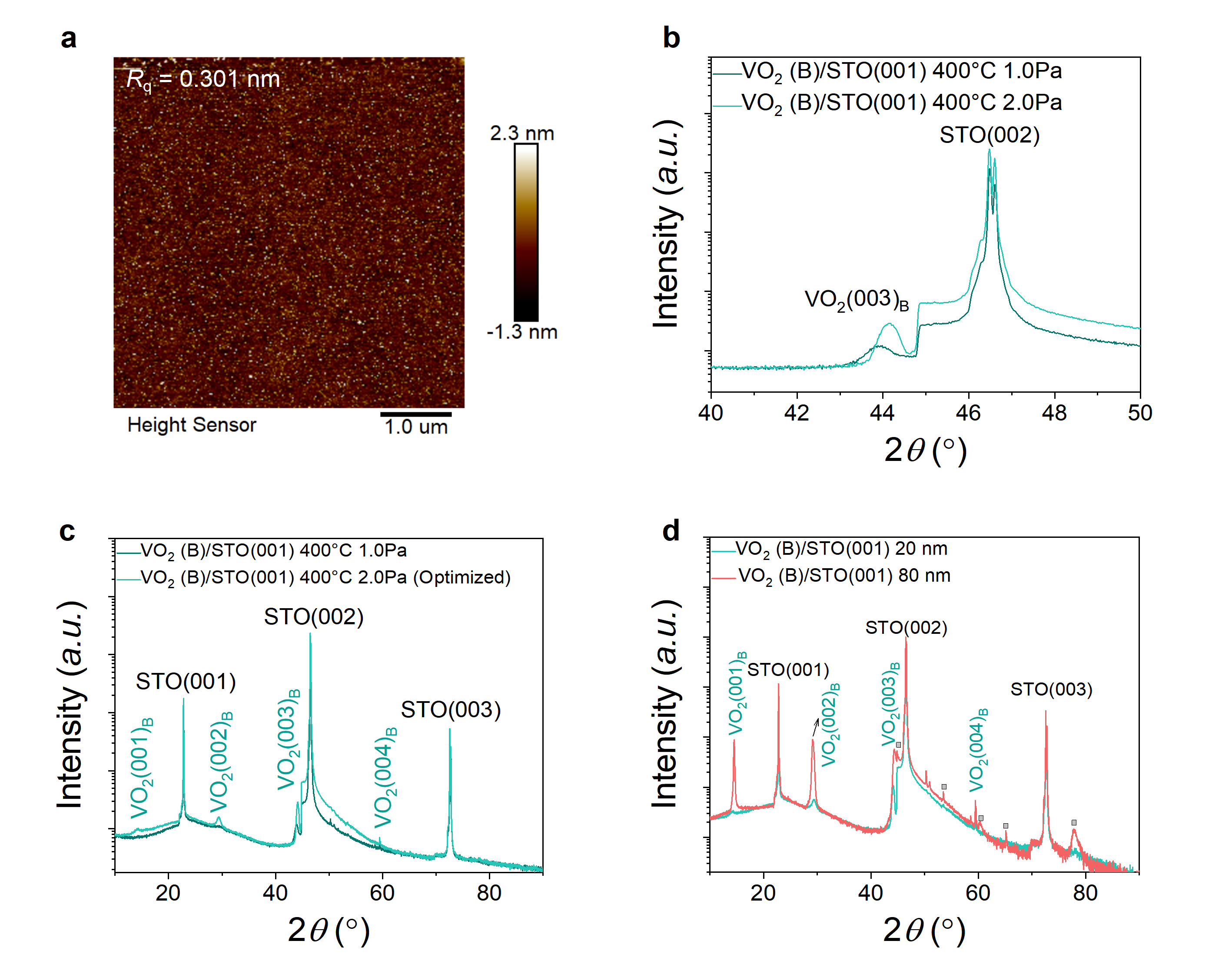


**Supplementary Figure 2. a**, Atomic force microscope (AFM) for as-deposited VO_2_ (B)/STO (001) heterostructure. **b**, X-ray diffraction (XRD) patterns as compared for VO_2_ (B)/STO (001) heterostructures upon various deposition conditions. **c**, X-ray diffraction (XRD) full spectra (from 10 ° to 90 °) as compared for VO_2_ (B)/STO (001) heterostructure. **d**, XRD spectra as compared for VO_2_ (B) films with different thickness. It can be seen that the root-mean-square roughness as achieved in metastable VO_2_ (B) is 0.301 nm, indicating a relatively smooth film surface. It is worthy to note that rutile VO_2_ (R) could appear within metastable VO_2_ (B) film via elevating the depositing temperature to a critical point of 430 °C, resulting in an unexpected mixed multiphase.^[26]^ Apart from the diffraction peak associated with the (003) plane of monoclinic VO_2_ (B), the characteristic peaks representing the (00*l*) plane for VO_2_ (B) are observed to be located at 14.40 °, 29.18°, and 59.48 °, indicating the preferential orientation associated with as-deposited VO_2_ (B) film.^[22]^ Further increasing the film thickness to 80 nm leads to the formation of the diffraction peaks related to the impurity phases in as-deposited VO_2_/STO (001) heterostructure (marked by the square). Therefore, an optimized growth condition (e.g., 400 °C, 2.0 Pa and 20 nm) was herein employed for depositing high-quality VO_2_ (B) film material.


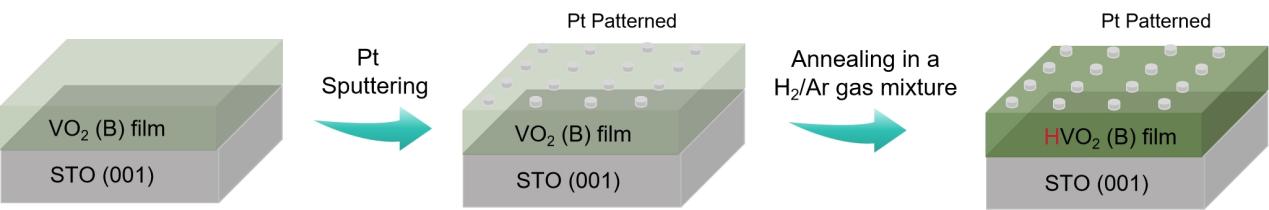


**Supplementary Figure 3.** Schematic of hydrogen spillover strategy for achieving the hydrogen evolution of VO_2_ (B) film. As a representative soft-chemistry approach,^[27]^ dot-shaped platinum as the catalyst were sputtered onto the as-deposited VO_2_ (B)/STO (001) heterostructure that reduces the required energy barrier for decomposing hydrogen modules deriving from H_2_/Ar gas mixture into hydrogen atoms. Afterwards, assisted by the elevated temperature (e.g., 100-300 °C), hydrogen atoms are further transformed into protons and electrons at the triple phase boundary that are further intercalated into the lattice of metastable VO_2_ (B) for achieving hydrogen evolution.

**Supplementary Table 1. Hydrogenation kinetics for VO_2_ using hydrogen spillover strategy**

| Sample | Temperature | Period | Electronic Phase | Refs. |
| --- | --- | --- | --- | --- |
| VO_2_ (M1) | 100 °C | 3 h | Electron-localized state | ^[11]^ |
| VO_2_ (M1) | 300 °C | 1 h | Electron-itinerant state | ^[28]^ |
| VO_2_ (B) | 100 °C | 3 h | Electron-itinerant state | This work |
| VO_2_ (B) | 300 °C | 1 h | Electron-itinerant state | This work |


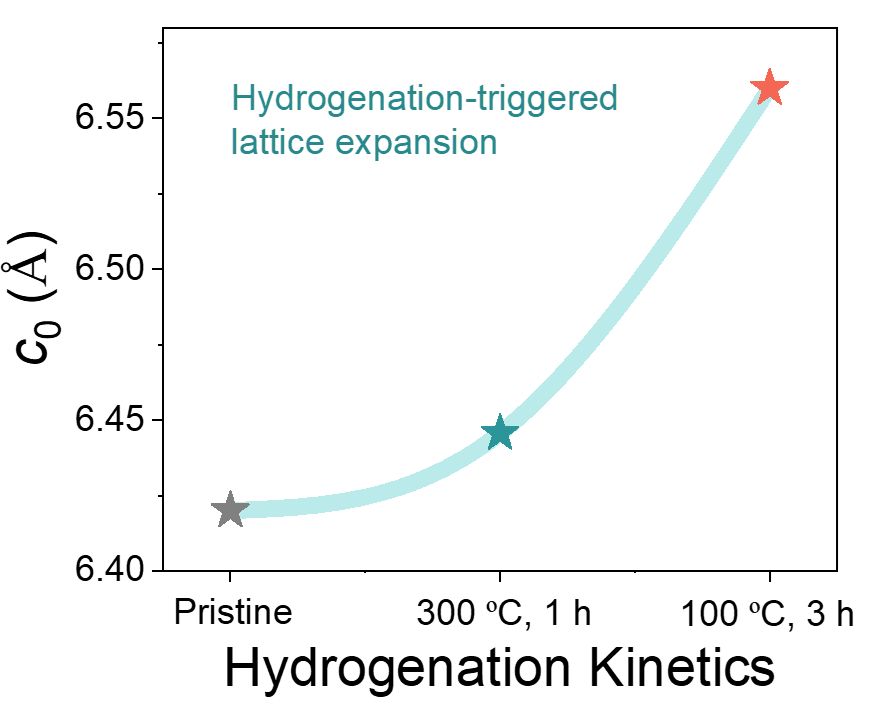


**Supplementary Figure 4.** The variation in the *cross-plane* lattice constant (e.g., *c*_0_) for VO_2_ (B) film before and after hydrogenation. Performing the hydrogenation triggers the *out-of-plane* lattice expansion of VO_2_ (B) film, owing to the formation of O-H interaction. Besides, the mild hydrogenation (e.g., 100 °C, 3 h) is prone to induce a larger lattice expansion.


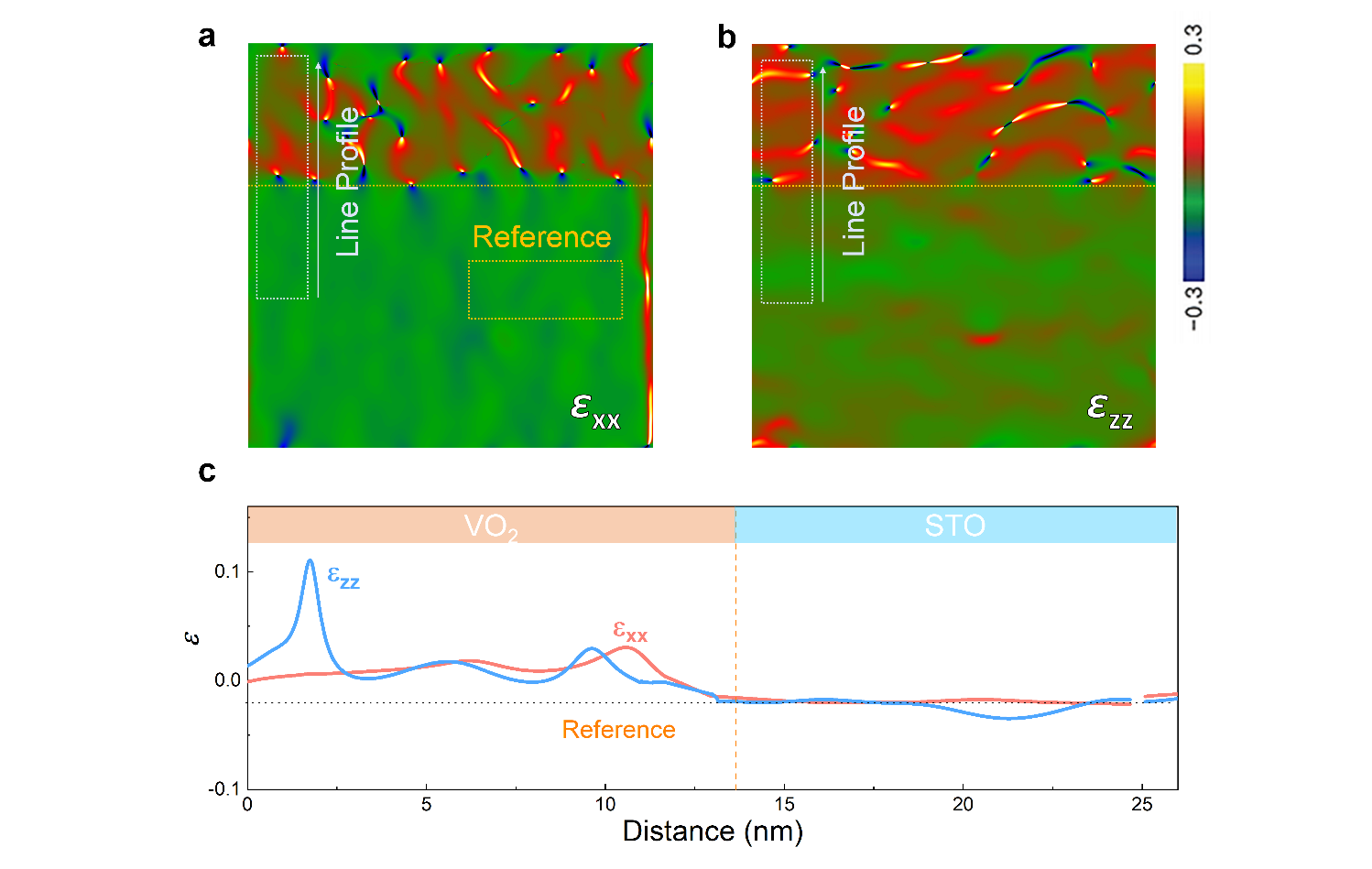


**Supplementary Figure 5.** The maps of **a**, *in-plane* and **b**, *out-of-plane* lattice strain (*ɛ*) as calculated from HAADF-STEM images of VO_2_/STO heterostructure by using the geometric phase analysis (GPA). **c**, Line profiles of *ɛ* as extracted from the lattice strain maps. Consistent with the aforementioned reciprocal space mapping (RSM) results, the metastable VO_2_ (B) film is epitaxially grown on the single crystalline STO (001) substrate. In addition, noting that the *in-plane* lattice of metastable VO_2_ (B) is locked by STO substrate, the *in-plane* lattice strain within hydrogenated VO_2_ (B) as calculated by using GPA analysis is lower than the one along the *out-of-plane* direction. Analogous to the RSM result (Figure 1d,e), incorporating protons into the lattice of VO_2_ (B) results in the anisotropy in the structural evolution, in which the *in-plane* lattice is locked by epitaxial STO substrate accompanied by a constraint-free *out-of-plane* lattice expansion via hydrogenation.


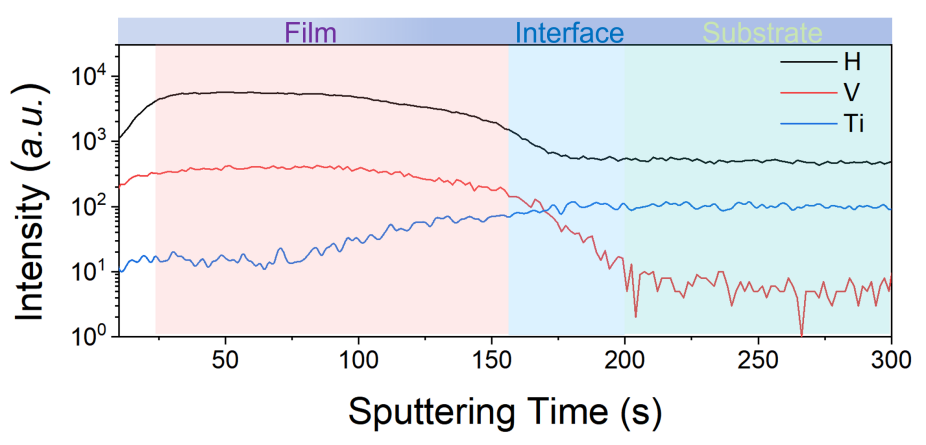


**Supplementary Figure 6.** Time-of-flight secondary-ion mass spectrometry (TOF-SIMS) measurement for VO_2_ (B) as hydrogenated at 100 ºC for 3 h.

**Supplementary Note 2. Chemical stability for hydrogenated VO_2_ (B) phase**

Seeking for the reversible but robust hydrogenated phases within electron-correlated system is a focal point for developing proton-based iontronic/ correlated electronic devices. Nevertheless, the chemical stability of hydrogenated correlated oxides is diverse, depending on specific material system. As a representative case, metallic hydrogenated phase of VO_2_ (M1) ^[12]^ and insulating hydrogenated phase of *Re*NiO_3_ ^[29]^ are rather stable upon the ambient atmosphere, which requires a high-temperature oxidizing annealing process to be recovered towards pristine state. In stark contrast, hydrogen-triggered electron-localized state of VO_2_ (M1) is unstable upon exposing to the air, in which situation the material resistivity and metal-insulator transition property are revived within ten hours.^[28]^ Although an enlarged Δ*G* of VO_2_ (B) determinates its intrinsically thermodynamic metastability, electron-itinerant hydrogenated phase of VO_2_ (B) is rather robust upon the ambient atmosphere within a month (Figure S7b), but can be reversibly recovered toward the initial state when annealing in a O_2_ atmosphere at 200 °C for 1 h. Further elevating the annealing temperature leads to the decomposition of hydrogenated VO_2_ (B). Therefore, the robust but reversible electron-itinerant state as newly discovered in the hydrogen-related phase diagram of metastable VO_2_ (B) holds great promise for developing new iontronic device applications.


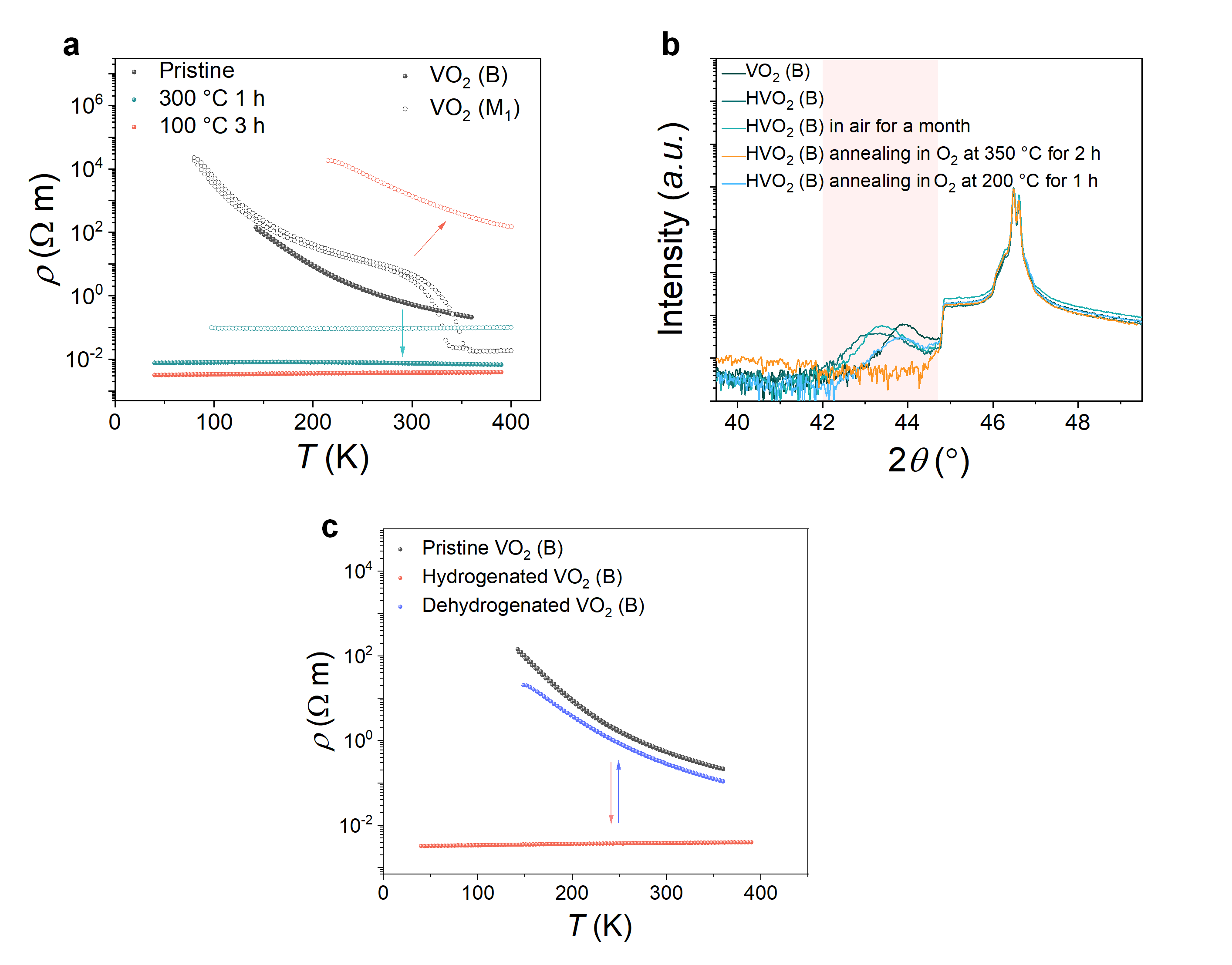


**Supplementary Figure 7. a**, Temperature dependence of material resistivity (*ρ*-*T*) as measured for the VO_2_ (B)/STO heterostructure upon various hydrogenation conditions, and also compared to the hydrogenated VO_2_ (M_1_) from ref ^[28]^. **b**, Variation in the XRD spectra of VO_2_ (B) upon the dehydrogenation process. **c**, Temperature dependence of material resistivity (*ρ*-*T*) as measured for the VO_2_ (B)/STO heterostructure upon hydrogenation and dehydrogenation process. It can be seen that performing the hydrogenation at whether 300 °C for 1 h or 100 °C for 3 h all triggers the formation of electron-itinerant state, without the electron localization being observed. By strong contrast, performing the low-temperature hydrogenation (e.g., 100 °C) for a long period (e.g., 3 h) under a H_2_/Ar gas mixture triggers the emergence of electron-localized hydrogenated VO_2_ (M_1_). In addition, such the hydrogen-triggered semiconductor-metal transition in metastable VO_2_ (B) is reversible via annealing at an oxygen-rich atmosphere, but robust upon exposing to the ambient atmosphere.


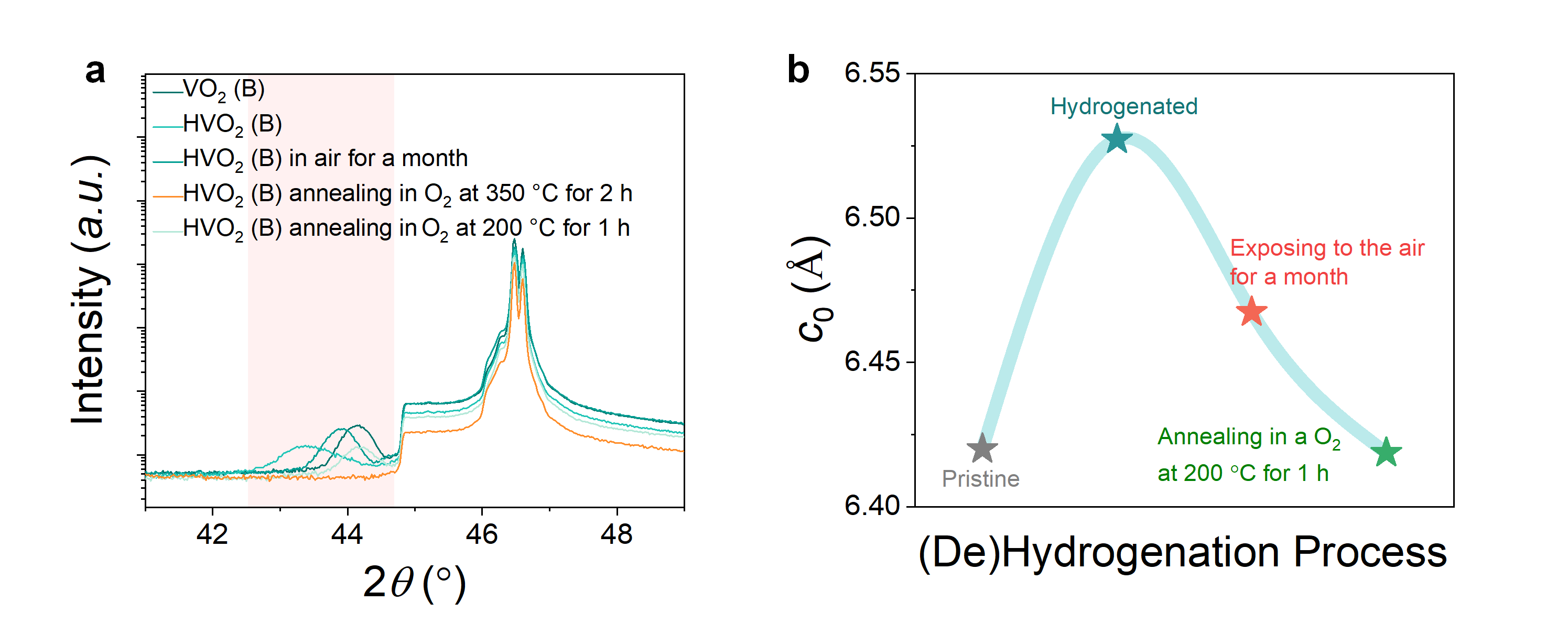


**Supplementary Figure 8. a**, Additional variation in the XRD spectra of VO_2_ (B) upon the dehydrogenation process. **b**, Additional variation in the *cross-plane* lattice constant (e.g., *c*_0_) for VO_2_ (B) film upon (de)hydrogenation process. Further altering the as-used VO_2_ (B) sample demonstrates the reproducibility of such hydrogen-associated topotactic transition within metastable VO_2_ (B).

**Supplementary Note 3. DFT calculations for hydrogenated VO_2_ (B).**

To investigate the influence of hydrogen absorption on the metastable VO_2_ (B), we inserted one hydrogen atom to any of the non-equivalent oxygen atoms in a 1×1×1 VO_2_ (B) unit cell while keeping the same Hubbard *U* of 3.8 eV. In order to obtain the particular hydrogen absorption energy, the atoms in the unit cell were fully relaxed for each configuration (Figure S9). Hydrogen adsorption is more energetically favorable along the empty *a*-axis direction of the monoclinic VO_2_ (B) (Figure 3d), e.g., H-1, H-3, H-12, and H-14 sites, similar to the preferentially diffused *a*_M_ (*c*_R_)-axis of VO_2_ (M1). In stark contrast, hydrogen adsorption is less favorable at the H-5, H-16, and H-17 sites, aligned along the *c*-axis direction of VO_2_ (B). In addition, the intermediate hydrogen absorption energies are observed for the case of adding hydrogen atoms along the *b*-axis direction, such as H-4 and H-8 sites. This understanding can be clearly demonstrated by comparing the hydrogen absorption at the O-1 and O-10 positions along different directions (e.g., H-1 site and H-2 site, H-14 site and H-15 site), as marked by blue in Figure 3c. Notably, as the band structure and DOS of hydrogenated VO_2_ (B) shown in Figures S11-S15, performing the hydrogenation process results in the finite DOS near the *E_F_* deriving from the spin-up electrons in the V-3*d* orbital that results in the formation of electron-itinerant state via proton evolution.


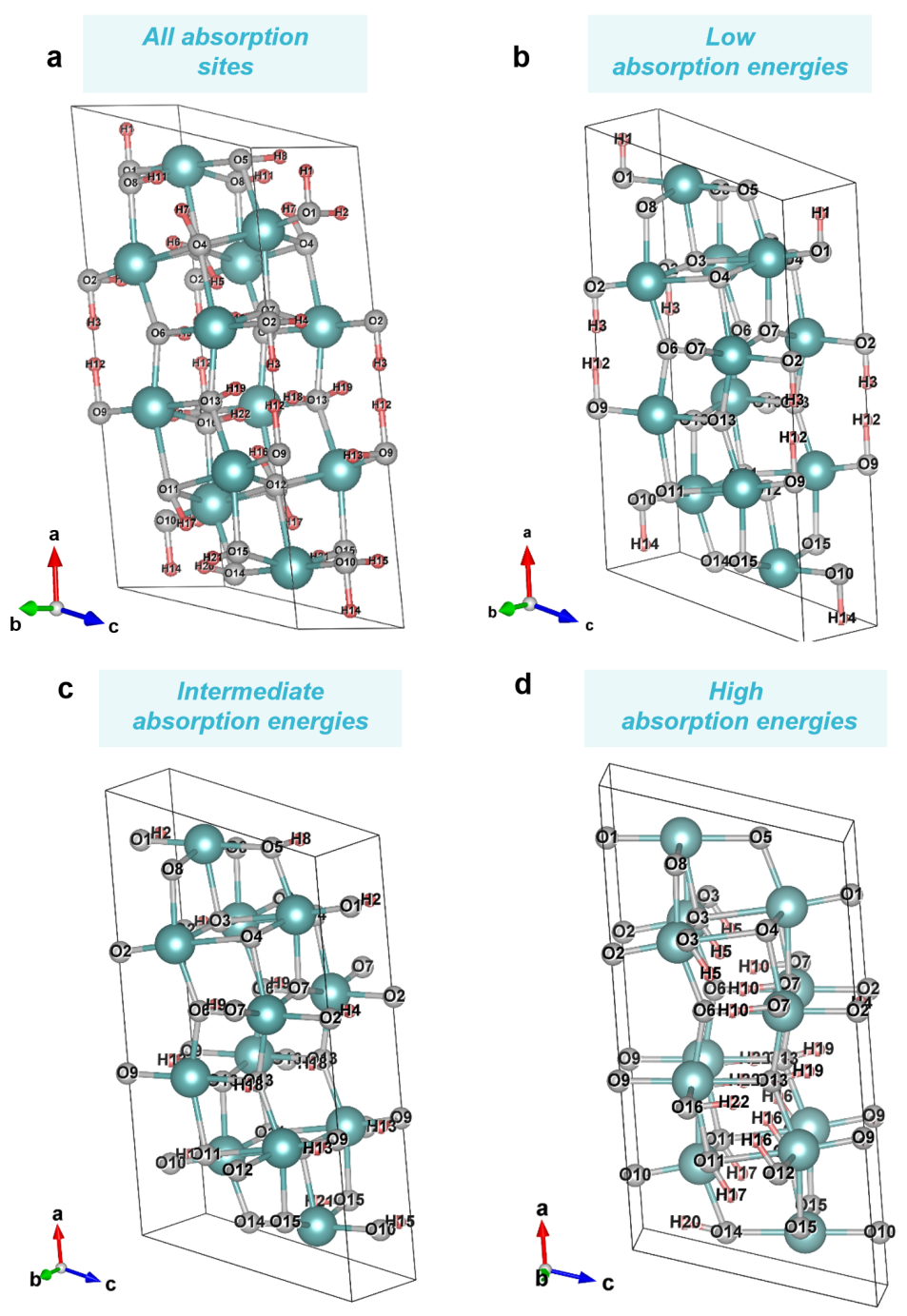


**Supplementary Figure 9. a**, Schematic of all the possible hydrogen absorption sites within the lattice of VO_2_ (B) using first-principles-based simulations. The hydrogen absorption in VO_2_ (B) with **b**, low absorption energy **c**, intermediate absorption energy and **d**, high absorption energy.


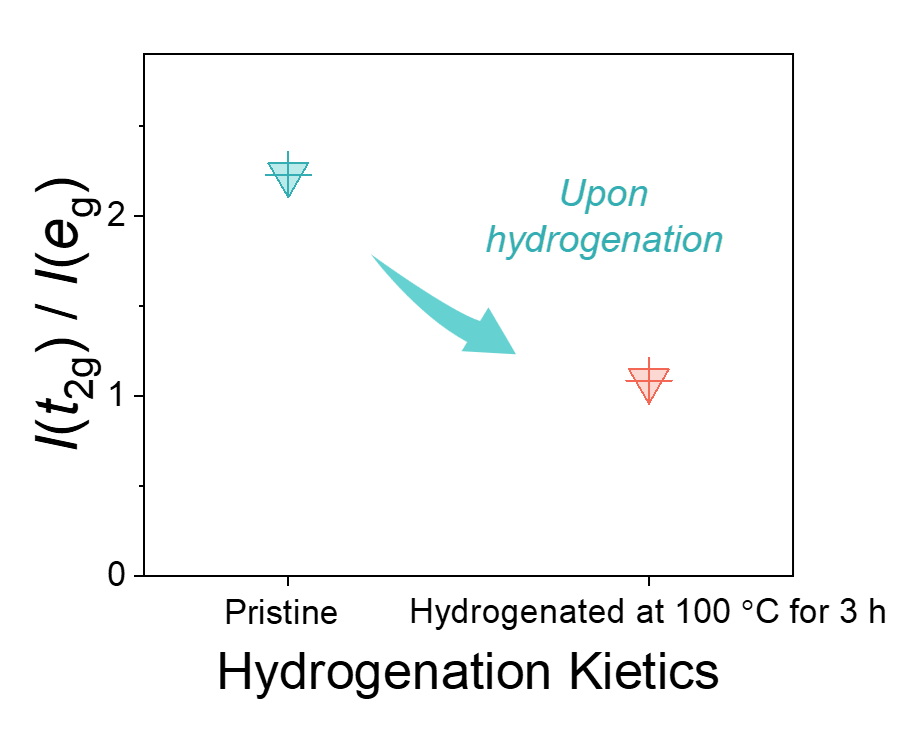


**Supplementary Figure 10.** Variation in the relative peak intensity associated to the *t*_2g_ and *e*_g_ orbital for VO_2_ (B) upon hydrogenation. Assisted by the NEXAFS analysis, the abundant electrons released by hydrogenation tend to occupy the low-energy *t*_2g_ orbital, reconfiguring the band structure of metastable VO_2_ (B).


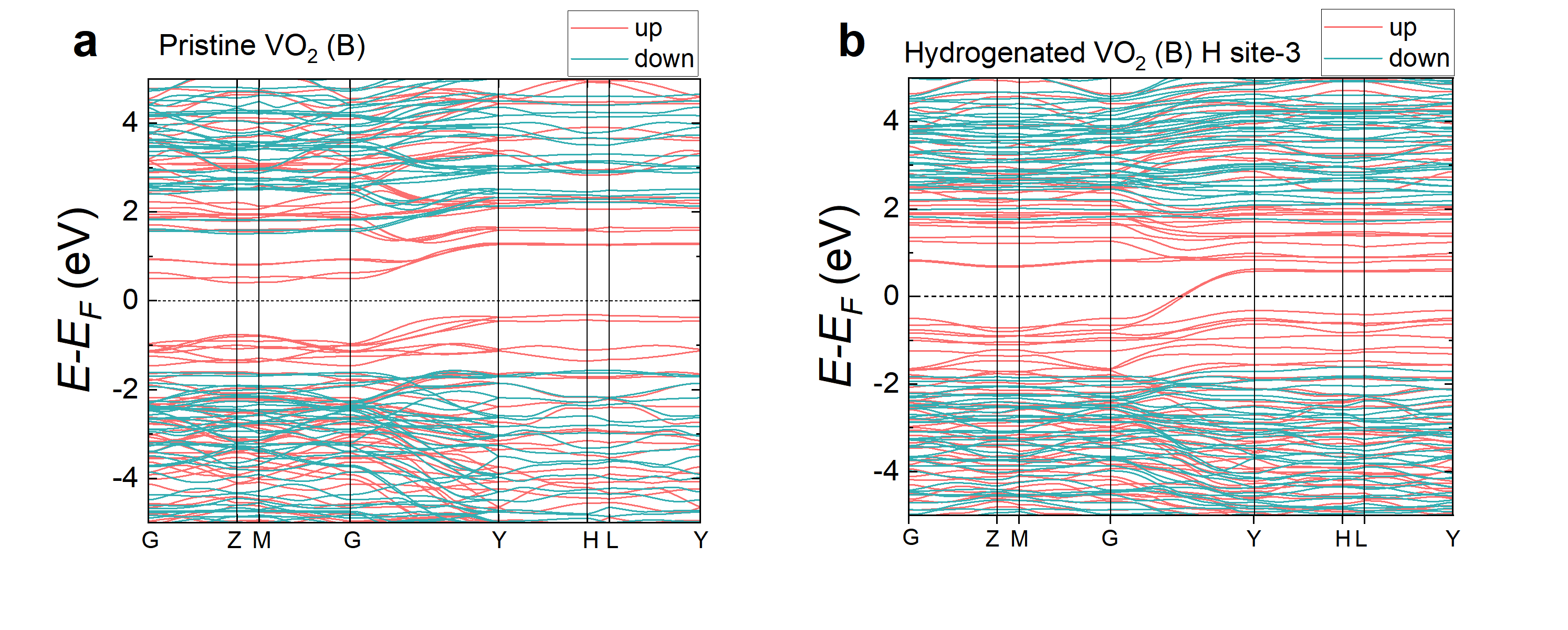


**Supplementary Figure 11.** Calculated band structure for **a**, pristine and **b**, hydrogenated VO_2_ (B) film using first-principles calculations.


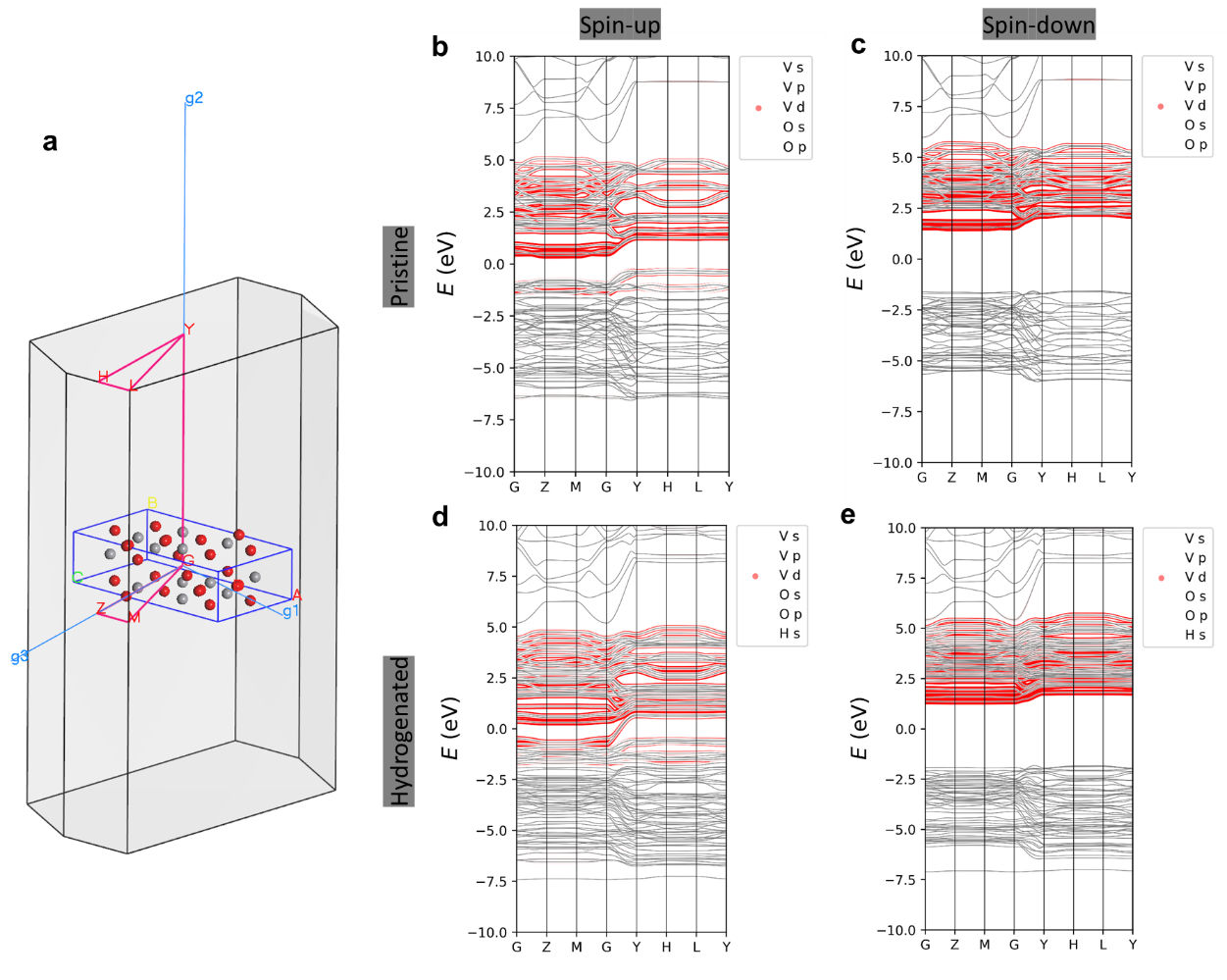


**Supplementary Figure 12. a**, Schematic of the first Brillouin zone for VO_2_ (B). The calculated band structure of **b**, spin-up VO_2_ (B) and **c**, spin-down VO_2_ (B) prior to hydrogenation; **d**, spin-up VO_2_ (B) and **e**, spin-down VO_2_ (B) upon hydrogenation using first-principles-based calculations.


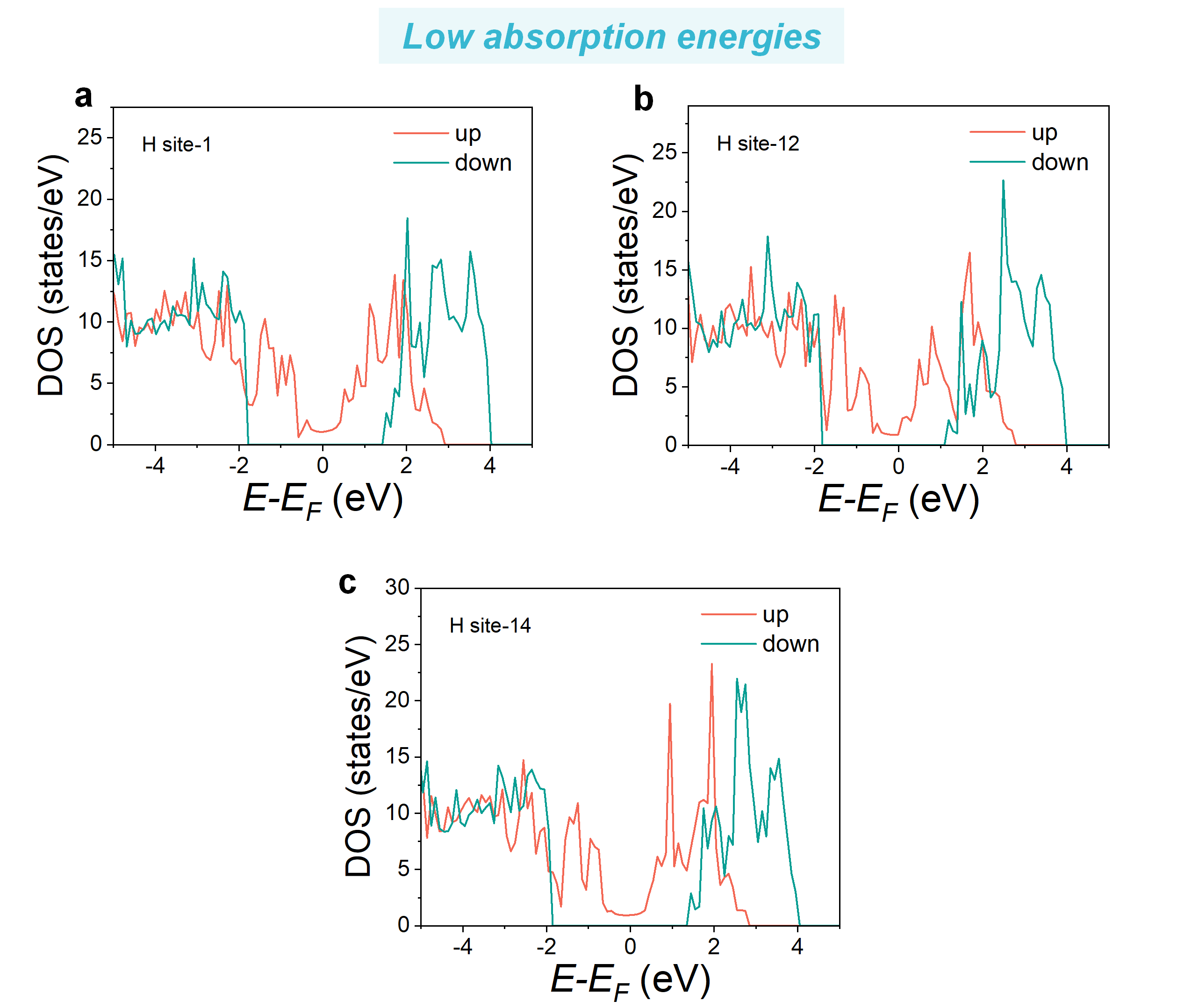


**Supplementary Figure 13.** Calculated density of states (DOS) of hydrogenated VO_2_ (B) at the **a**, H-1 site, **b** H-12 site and **c**, H-14 site using first-principles calculations.


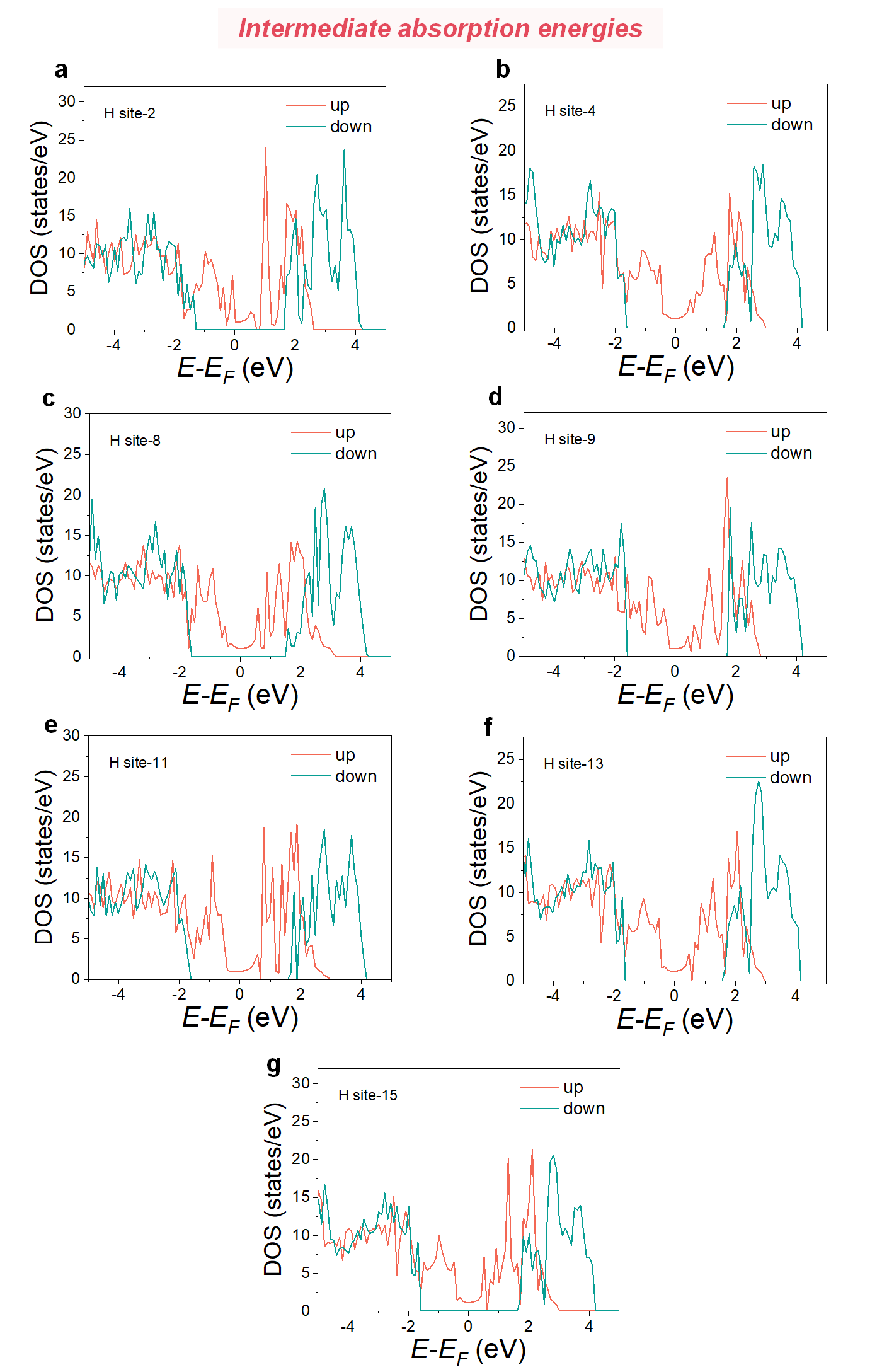


**Supplementary Figure 14.** Calculated DOS of hydrogenated VO_2_ (B) at the **a**, H-2 site, **b** H-4 site, **c**, H-8 site, **d**, H-9 site, **e**, H-11 site, **f**, H-13 site, and **g**, H-15 using first-principles-based calculations.


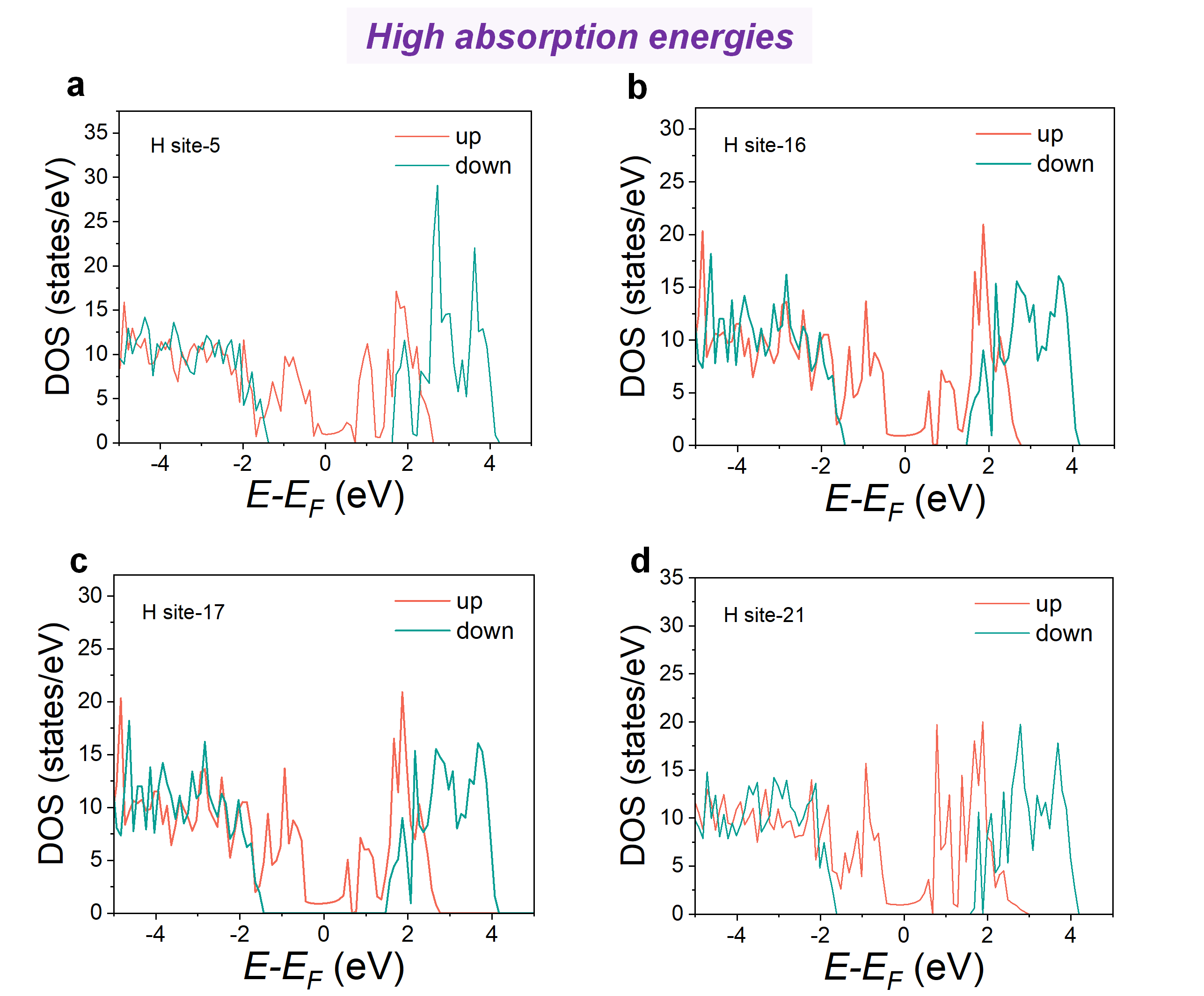


**Supplementary Figure 15.** Calculated DOS of hydrogenated VO_2_ (B) at the **a**, H-5 site, **b** H-16 site, **c**, H-17 and **d**, H-21 site using first-principles-based calculations.

**Supplementary Note 4. Hydrogen-associated phase modulation within metastable VO_2_ (A)**

Here we further extend the proton evolution to metastable VO_2_ (A) polymorph that exhibits a tetragonal crystalline structure and less distorted VO_6_ octahedron. Utilizing the density functional theory (DFT) calculations,^[30]^ the forming energy (Δ*G*) for metastable VO_2_ (A) phase was previously revealed to be smaller than that of metastable VO_2_ (B), indicating a relatively robust crystal structure. Considering the similar *a*-axis lattice constant of VO_2_ (A) ($\frac{\sqrt{2}}{3}$*a*_0, film_ = 3.978 Å) and STO (*a*_0, sub._ = 3.905 Å), metastable VO_2_ (A) films are deposited onto the (011)-oriented single crystalline STO substrate as epitaxial template. As the X-ray diffraction (XRD) results shown in Figure S16a, the diffraction peak associated with the (600) plane of VO_2_ (A) (e.g., 67.6 °) just locates at the shoulder associated with the (022) plane of STO substrate located at 67.8 °. In addition, the electrical transport property for metastable VO_2_ (A) is similar to the one for typical insulator (Figure S16b), and the resistive magnitude of which is enlarged with respective to the VO_2_ (B) and VO_2_ (M1), consistent with previous report.^[24]^ Analogous hydrogen spillover strategy was employed to achieve effective hydrogen intercalation in metastable VO_2_ (A), resulting in a slight lattice expansion and insulator-metal transition (Figure S17). Similar to metastable VO_2_ (B), performing the hydrogenation at 100 °C for 3 h results in a more abrupt variation in the material resistivity (e.g., *R*_0_/*R*_H_) of metastable VO_2_ (A) phase (Figure S18). Accompanied by the electronic phase transition, the valence state of vanadium for hydrogenated VO_2_ (A) is reduced from +4 to +3, while the introduced hydrogens tend to bond with the lattice oxygen to form such the O-H interactions in the O 1*s* spectrum (Figure S19). Hydrogenation-introduced electrons are expected to occupy the low-energy empty *t*_2g_ orbital, resulting in such the hydrogen-related Mott transition in metastable VO_2_ (A) toward a new metallic hydrogenated state. Introducing non-equilibrium conditions to electron-correlated system is envisioned to be a universal strategy to access exotic electron phases and functionality that do not exist in equilibrium phase diagram. In addition to thermodynamically stable material system, one remaining challenge lies in an intrinsic metastability that easily disturbs the expected crystalline structure upon hydrogenation, rather than triggering a topotactic transition. Therefore, a promising candidate in metastable material system for achieving proton evolution requires to obtain a suitable Δ*G* and/or open lattice framework. The highly robust but reversible phase modulations herein achievable in metastable VO_2_ through hydrogenation are the proof-of-principle demonstration of potential protonic device applications in the field of correlated electronic, artificial intelligence and energy conversions. For example, on the basis of hydrogenated metastable VO_2_, three-terminal electrochemical transistor can be fabricated, in which the liquid or solid-state proton source can be utilized as electrolyte to reversibly inject the protons into the metastable VO_2_ channel for achieving the electrically tunable resistive switching. Analogous to hydrogenated VO_2_ (M1) ^[31]^, such the newly discovered metallic hydrogenated phases in metastable VO_2_ system are expected to make it as the neuromorphic material to imitate human brain’s functionality (e.g., neuron and synapse). Combined with a special open framework, metastable VO_2_ (B) could have great potential in the field of energy conversions (e.g., hybrid battery system). ^[32]^


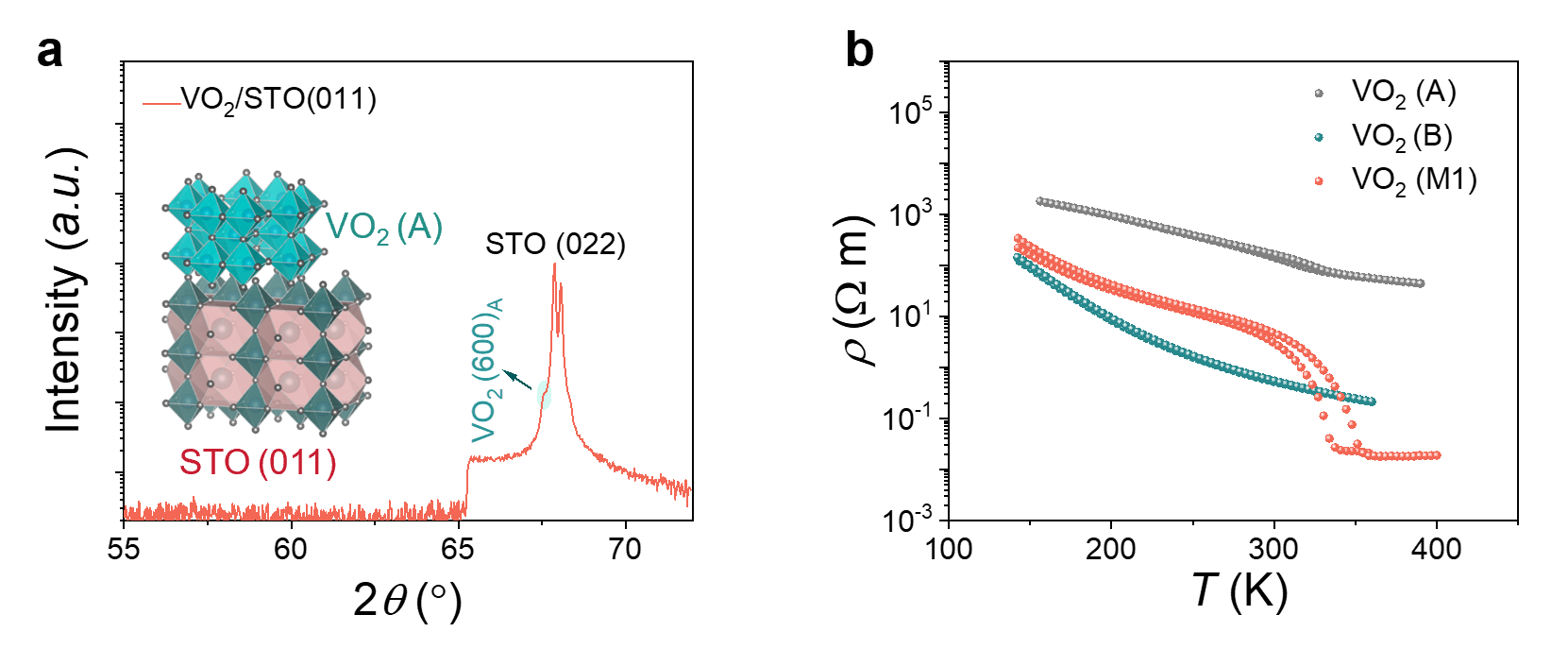


**Supplementary Figure 16. a**, The XRD spectra of VO_2_ (B)/STO (001) heterostructure. **b**, Temperature dependence of material resistivity (*ρ*-*T*) as measured for the VO_2_ (B)/STO heterostructure.


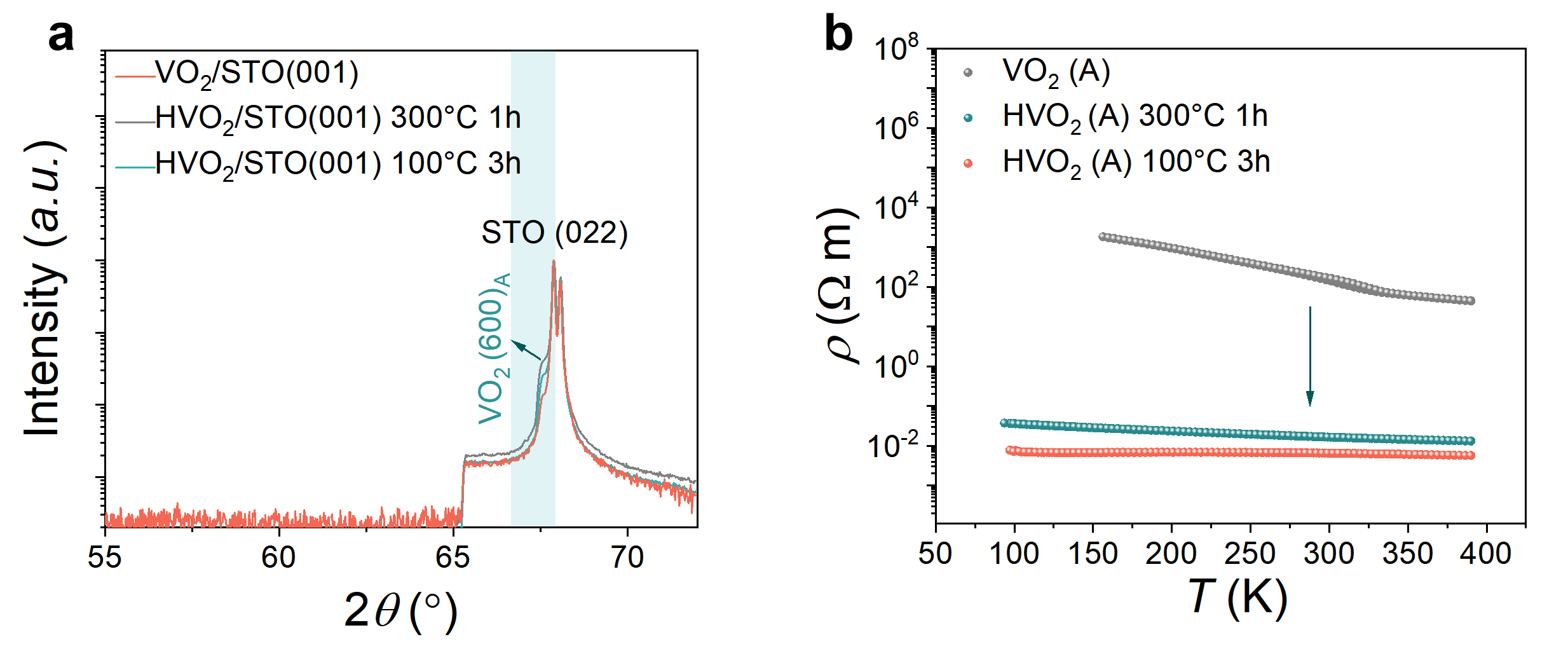


**Supplementary Figure 17. a**, X-ray diffraction (XRD) patterns as compared for VO_2_ (A)/STO (011) heterostructures upon various hydrogenation kinetics, while the configuration of VO_2_ (A)/STO (011) heterostructure is schematically illustrated in the inset. **b**, *ρ*-*T* tendencies as measured for the VO_2_ (A) films upon various hydrogenation conditions.


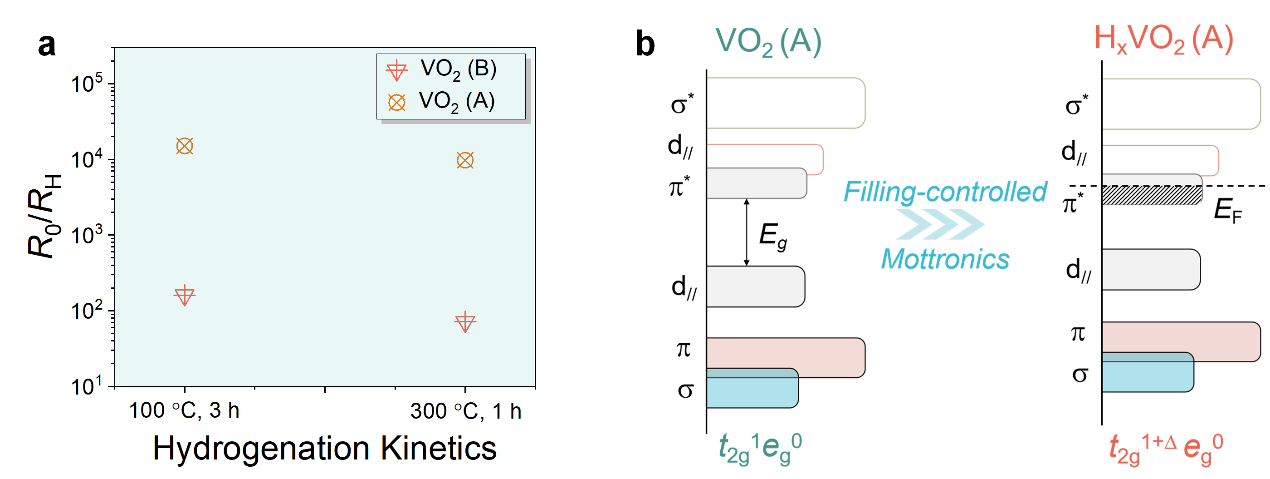


**Supplementary Figure 18. a**, Hydrogen-induced variation in the material resistivity (*R*_0_/*R*_H_) as compared for VO_2_ (B) and VO_2_ (A) via hydrogenation. **b**, Schematic of orbital reconfiguration of VO_2_ (A) via hydrogen evolution.


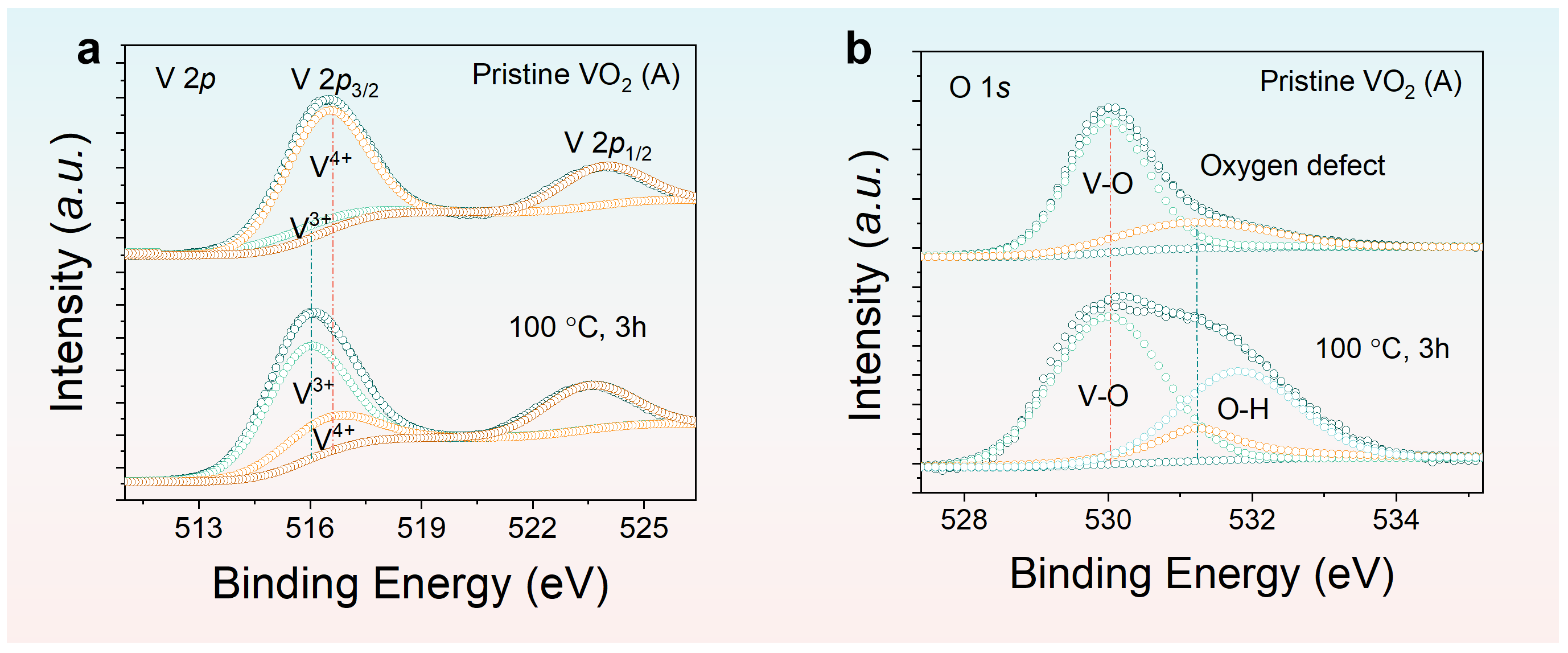


**Supplementary Figure 19.** The X-ray photoelectron spectra (XPS) for the core levels of **a**, vanadium and **b**, oxygen of VO_2_ (A)/STO (001) heterostructure as hydrogenated 100 ºC for 3 hours. Utilizing the XPS analysis, the valence state of vanadium for VO_2_ (A) is also reduced toward +3 via hydrogenation, with the formation of O-H interactions being observed.


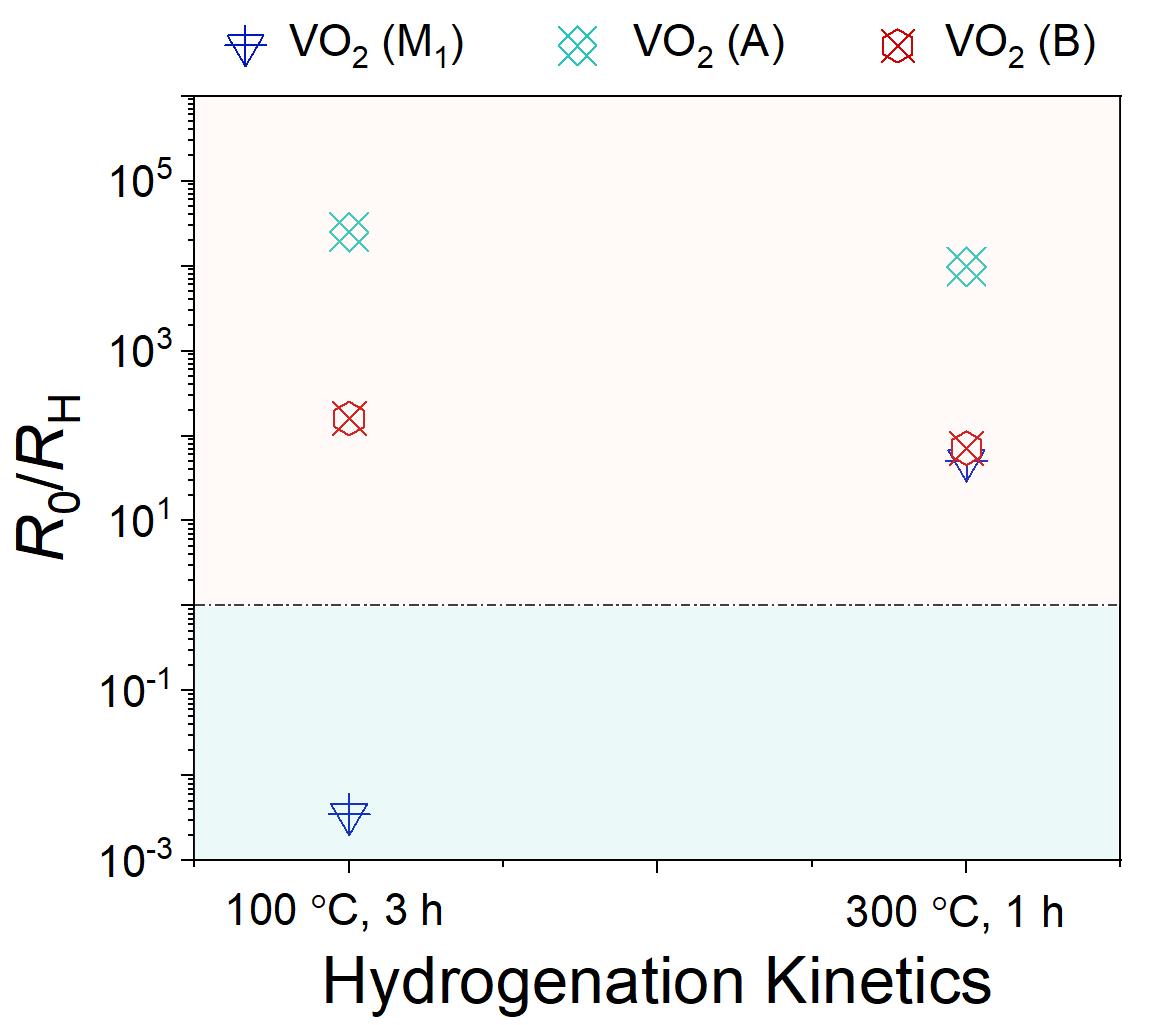


**Supplementary Figure 20.** Comparing the hydrogen-associated resistive regulation (*R*_0_/*R*_H_) for metastable VO_2_ and stable VO_2_ (M_1_).^[28]^

**Supplementary Table 2. Hydrogen-associated electronic phase transitions within transitional metal oxides.**

| Transitional metal oxides | Thermodynamic status | Hydrogenation conditions | Hydrogenated Phase | Resistive modulation (*R*_H_/*R*_0_) | Refs. |
| --- | --- | --- | --- | --- | --- |
| VO_2_ (M1) | Stable | Hydrogen spillover; 100 ºC 3h | Electron-localized state | 1×10^2^ | ^[11]^ |
| VO_2_ (M1) | Stable | Hydrogen spillover; 300 ºC 1h | Electron-itinerant state | 3×10^-1^ | ^[12]^ |
| VO_2_ (B) | Metastable | Hydrogen spillover; 100 ºC 3h/ 300 ºC 1h | Electron-itinerant state | 6×10^-3^ | This work |
| VO_2_ (A) | Metastable | Hydrogen spillover; 100 ºC 3h/ 300 ºC 1h | Electron-itinerant state | 3.5×10^-5^ | This work |
| Ni_1-x_O | Stable | Hydrogen spillover; 200 ºC 1h | Electron-localized state | 7×10^3^ | ^[33]^ |
| WO_3_ | Stable | Tip-induced hydrogenation;  room temperature | Electron-itinerant state | 1.6×10^-3^ | ^[34]^ |
| SrCoO_2.5_ | Stable | Ionic liquid gating;  room temperature | Electron-localized state | 7×10^1^ | ^[35]^ |
| NdNiO_3_ | Metastable | Hydrogen spillover; 100 ºC 1h | Electron-localized state | 1×10^3^ | ^[36]^ |
| ErNiO_3_ | Metastable | Hydrogen spillover; 100 ºC 1h | Decomposition | / | ^[36]^ |
| HoNiO_3_ | Metastable | Hydrogen spillover; 100 ºC 1h | Decomposition | / | ^[36]^ |
| ZnO | Stable | Ionic liquid gating;  room temperature | Electron-itinerant state | 1.4×10^-1^ | ^[37]^ |
| NiCo_2_O_4_ | Stable | Ionic liquid gating;  100 ºC | Electron-localized state | 2×10^5^ | ^[38]^ |

**Supplementary References**

[1] S. Shen, M. Wang, Y. Zhang, Y. Lyu, D. Tian, C. Gao, Y. Long, J. Zhao, P. Yu, Coexistence of Both Localized Electronic States and Electron Gas at Rutile TiO_2_ Surfaces. *Adv. Mater.* **2023**, 35, 2301453.

[2] S. Ning, S. C. Huberman, Z. Ding, H.-H. Nahm, Y.-H. Kim, H.-S. Kim, G. Chen, C. A. Ross, Anomalous Defect Dependence of Thermal Conductivity in Epitaxial WO_3_ Thin Films. *Adv. Mater.* **2019**, 31, 1903738.

[3] S. G. Altendorf, J. Jeong, D. Passarello, N. B. Aetukuri, M. G. Samant, S. S. P. Parkin, Facet-Independent Electric-Field-Induced Volume Metallization of Tungsten Trioxide Films. *Adv. Mater.* **2016**, 28, 5284.

[4] T. Su, P. C. Taylor, G. Ganguly, D. E. Carlson, Direct Role of Hydrogen in the Staebler-Wronski Effect in Hydrogenated Amorphous Silicon. *Phys. Rev. Lett.* **2002**, 89, 015502.

[5] C. G. Van de Walle, J. Neugebauer, Universal alignment of hydrogen levels in semiconductors, insulators and solutions. *Nature* **2003**, 423, 626.

[6] C. G. Van de Walle, Hydrogen as a Cause of Doping in Zinc Oxide. *Phys. Rev. Lett.* **2000**, 85, 1012.

[7] X. Zhou, H. Li, Y. Jiao, G. Zhou, H. Ji, Y. Jiang, X. Xu, Hydrogen‐Associated Multiple Electronic Phase Transitions for d‐Orbital Transitional Metal Oxides: Progress, Application, and Beyond. *Adv. Funct. Mater.* **2024**, 34, 2316536.

[8] J. Lee, Y. Ha, S. Lee, Hydrogen Control of Double Exchange Interaction in La_0.67_Sr_0.33_MnO_3_ for Ionic-Electric-Magnetic Coupled Applications. *Adv. Mater.* **2021**, 33, 2007606.

[9] X. C. Zhou, W. Mao, Y. C. Cui, H. Zhang, Q. Liu, K. Q. Nie, X. G. Xu, Y. Jiang, N. F. Chen, J. K. Chen, Multiple Electronic Phase Transitions of NiO via Manipulating the NiO_6_ Octahedron and Valence Control. *Adv. Funct. Mater.* **2023**, 33, 2303416.

[10] S. Choi, J. Son, J. L. MacManus-Driscoll, S. Lee, Hydrogen-Driven Low-Temperature Topotactic Transition in Nanocomb Cobaltite for Ultralow Power Ionic–Magnetic Coupled Applications. *Nano Lett.* **2024**, 24, 3606.

[11] H. Yoon, M. Choi, T. W. Lim, H. Kwon, K. Ihm, J. K. Kim, S. Y. Choi, J. Son, Reversible phase modulation and hydrogen storage in multivalent VO_2_ epitaxial thin films. *Nat. Mater.* **2016**, 15, 1113.

[12] J. Wei, H. Ji, W. H. Guo, A. H. Nevidomskyy, D. Natelson, Hydrogen stabilization of metallic vanadium dioxide in single-crystal nanobeams. *Nat. Nanotechnol.* **2012**, 7, 357.

[13] X. Deng, Y.-X. Liu, Z.-Z. Yang, Y.-F. Zhao, Y.-T. Xu, M.-Y. Fu, Y. Shen, K. Qu, Z. Guan, W.-Y. Tong, Y.-Y. Zhang, B.-B. Chen, N. Zhong, P.-H. Xiang, C.-G. Duan, Spatial evolution of the proton-coupled Mott transition in correlated oxides for neuromorphic computing. *Sci. Adv.* **2024**, 10, eadk9928.

[14] J. Park, H. Yoon, H. Sim, S. Y. Choi, J. Son, Accelerated Hydrogen Diffusion and Surface Exchange by Domain Boundaries in Epitaxial VO_2_ Thin Films. *ACS Nano* **2020**, 14, 2533.

[15] X. Zhou, H. Li, Y. Shang, F. Meng, Z. Li, K. Meng, Y. Wu, X. Xu, Y. Jiang, N. Chen, J. Chen, Manipulating the metal-to-insulator transitions of VO_2_ by combining compositing and doping strategies. *Phys. Chem. Chem. Phys.* **2023**, 25, 21908.

[16] X. Zhou, Y. Cui, Y. Shang, H. Li, J. Wang, Y. Meng, X. Xu, Y. Jiang, N. Chen, J. Chen, Non-equilibrium Spark Plasma Reactive Doping Enables Highly Adjustable Metal-to-Insulator Transitions and Improved Mechanical Stability for VO_2_. *J. Phys. Chem. C* **2023**, 127, 2639.

[17] Z. Zhang, F. Zuo, C. H. Wan, A. Dutta, J. Kim, J. Rensberg, R. Nawrodt, H. H. Park, T. J. Larrabee, X. F. Guan, Y. Zhou, S. M. Prokes, C. Ronning, V. M. Shalaev, A. Boltasseva, M. A. Kats, S. Ramanathan, Evolution of Metallicity in Vanadium Dioxide by Creation of Oxygen Vacancies. *Phys. Rev. Appl.* **2017**, 7.

[18] J. K. Chen, Y. Zhou, S. Middey, J. Jiang, N. F. Chen, L. D. Chen, X. Shi, M. Dobeli, J. Shi, J. Chakhalian, S. Ramanathan, Self-limited kinetics of electron doping in correlated oxides. *Appl. Phys. Lett.* **2015**, 107, 031905.

[19] F. Théobald, R. Cabala, J. Bernard, Essai sur la structure de VO_2_(B). *J. Solid State Chem.* **1976**, 17, 431.

[20] S. R. Popuri, M. Miclau, A. Artemenko, C. Labrugere, A. Villesuzanne, M. Pollet, Rapid Hydrothermal Synthesis of VO_2_ (B) and Its Conversion to Thermochromic VO_2_ (M1). *Inorg. Chem.* **2013**, 52, 4780.

[21] P. Hu, P. Hu, T. D. Vu, M. Li, S. Wang, Y. Ke, X. Zeng, L. Mai, Y. Long, Vanadium Oxide: Phase Diagrams, Structures, Synthesis, and Applications. *Chem. Rev.* **2023**, 123, 4353.

[22] A. Chen, Z. Bi, W. Zhang, J. Jian, Q. Jia, H. Wang, Textured metastable VO_2_ (B) thin films on SrTiO_3_ substrates with significantly enhanced conductivity. *Appl. Phys. Lett.* **2014**, 104.

[23] S. Lee, X.-G. Sun, A. A. Lubimtsev, X. Gao, P. Ganesh, T. Z. Ward, G. Eres, M. F. Chisholm, S. Dai, H. N. Lee, Persistent Electrochemical Performance in Epitaxial VO_2_(B). *Nano Lett.* **2017**, 17, 2229.

[24] A. Srivastava, H. Rotella, S. Saha, B. Pal, G. Kalon, S. Mathew, M. Motapothula, M. Dykas, P. Yang, E. Okunishi, D. D. Sarma, T. Venkatesan, Selective growth of single phase VO_2_(A, B, and M) polymorph thin films. *APL Mater.* **2015**, 3, 026101.

[25] S. Lee, T. L. Meyer, C. Sohn, D. Lee, J. Nichols, D. Lee, S. S. A. Seo, J. W. Freeland, T. W. Noh, H. N. Lee, Electronic structure and insulating gap in epitaxial VO_2_ polymorphs. *APL Mater.* **2015**, 3, 126109.

[26] S. Lee, I. N. Ivanov, J. K. Keum, H. N. Lee, Epitaxial stabilization and phase instability of VO_2_ polymorphs. *Sci. Rep.* **2016**, 6, 19621.

[27] L. Li, M. Wang, Y. Zhou, Y. Zhang, F. Zhang, Y. Wu, Y. Wang, Y. Lyu, N. Lu, G. Wang, H. Peng, S. Shen, Y. Du, Z. Zhu, C.-W. Nan, P. Yu, Manipulating the insulator-metal transition through tip-induced hydrogenation. *Nat. Mater.* **2022**, 21, 1246.

[28] X. Zhou, H. Li, F. Meng, W. Mao, J. Wang, Y. Jiang, K. Fukutani, M. Wilde, B. Fugetsu, I. Sakata, N. Chen, J. Chen, Revealing the Role of Hydrogen in Electron-Doping Mottronics for Strongly Correlated Vanadium Dioxide. *J. Phys. Chem. Lett.* **2022**, 13, 8078.

[29] J. Shi, Y. Zhou, S. Ramanathan, Colossal resistance switching and band gap modulation in a perovskite nickelate by electron doping. *Nat. Commun.* **2014**, 5, 4860.

[30] S. D. Zhang, B. Shang, J. L. Yang, W. S. Yan, S. Q. Wei, Y. Xie, From VO_2_ (B) to VO_2_ (A) nanobelts: first hydrothermal transformation, spectroscopic study and first principles calculation. *Phys. Chem. Chem. Phys.* **2011**, 13, 15873.

[31] S. Deng, H. Yu, T. J. Park, A. N. M. N. Islam, S. Manna, A. Pofelski, Q. Wang, Y. Zhu, S. K. R. S. Sankaranarayanan, A. Sengupta, S. Ramanathan, Selective area doping for Mott neuromorphic electronics. *Sci. Adv.* **2023**, 9, eade4838.

[32] Y. Li, Q. Zhang, Y. Yuan, H. Liu, C. Yang, Z. Lin, J. Lu, Surface Amorphization of Vanadium Dioxide (B) for K-Ion Battery. *Adv. Energy Mater.* **2020**, 10, 2000717.

[33] Z. Zhang, S. Mondal, S. Mandal, J. M. Allred, N. A. Aghamiri, A. Fali, Z. Zhang, H. Zhou, H. Cao, F. Rodolakis, J. L. McChesney, Q. Wang, Y. Sun, Y. Abate, K. Roy, K. M. Rabe, S. Ramanathan, Neuromorphic learning with Mott insulator NiO. *P Natl. Acad. Sci. USA* **2021**, 118, e2017239118.

[34] F. Zhang, Y. Zhang, L. L. Li, X. Mou, H. N. Peng, S. C. Shen, M. Wang, K. H. Xiao, S. H. Ji, D. Yi, T. X. Nan, J. S. Tang, P. Yu, Nanoscale multistate resistive switching in WO_3_ through scanning probe induced proton evolution. *Nat. Commun.* **2023**, 14, 3950.

[35] N. P. Lu, P. F. Zhang, Q. H. Zhang, R. M. Qiao, Q. He, H. B. Li, Y. J. Wang, J. W. Guo, D. Zhang, Z. Duan, Z. L. Li, M. Wang, S. Z. Yang, M. Z. Yan, E. Arenholz, S. Y. Zhou, W. L. Yang, L. Gu, C. W. Nan, J. Wu, Y. Tokura, P. Yu, Electric-field control of tri-state phase transformation with a selective dual-ion switch. *Nature* **2017**, 546, 124.

[36] Y. Bian, H. Y. Li, F. B. Yan, H. F. Li, J. U. Wang, H. Zhang, Y. Jiang, N. F. Chen, J. K. Chen, Hydrogen induced electronic transition within correlated perovskite nickelates with heavy rare-earth composition. *Appl. Phys. Lett.* **2022**, 120, 092103.

[37] H. T. Yuan, H. Shimotani, J. T. Ye, S. Yoon, H. Aliah, A. Tsukazaki, M. Kawasaki, Y. Iwasa, Electrostatic and Electrochemical Nature of Liquid-Gated Electric-Double-Layer Transistors Based on Oxide Semiconductors. *J. Am. Chem. Soc.* **2010**, 132, 18402.

[38] M. Wang, X. L. Sui, Y. J. Wang, Y. H. Juan, Y. J. Lyu, H. N. Peng, T. T. Huang, S. C. Shen, C. G. Guo, J. B. Zhang, Z. L. Li, H. B. Li, N. P. Lu, A. T. N'Diaye, E. Arenholz, S. Y. Zhou, Q. He, Y. H. Chu, W. H. Duan, P. Yu, Manipulate the Electronic and Magnetic States in NiCo_2_O_4_ Films through Electric-Field-Induced Protonation at Elevated Temperature. *Adv. Mater.* **2019**, 31, 1900458.
